# Sensory integration dynamics in a hierarchical network explains choice probabilities in cortical area MT

**DOI:** 10.1038/ncomms7177

**Published:** 2015-02-04

**Authors:** Klaus Wimmer, Albert Compte, Alex Roxin, Diogo Peixoto, Alfonso Renart, Jaime de la Rocha

**Affiliations:** 1 Institut d'Investigacions Biomèdiques August Pi i Sunyer (IDIBAPS), C/Rosselló 149, 08036 Barcelona, Spain; 2 Centre de Recerca Matemàtica (CRM), Campus de Bellaterra, Edifici C, 08193 Barcelona, Spain; 3 Champalimaud Neuroscience Programme, Champalimaud Centre for the Unknown, 1400-038 Lisbon, Portugal; 4 Department of Neurobiology, Stanford University, 299 Campus Drive West , Stanford, California 94305-5125, USA

## Abstract

Neuronal variability in sensory cortex predicts perceptual decisions. This relationship, termed choice probability (CP), can arise from sensory variability biasing behaviour and from top-down signals reflecting behaviour. To investigate the interaction of these mechanisms during the decision-making process, we use a hierarchical network model composed of reciprocally connected sensory and integration circuits. Consistent with monkey behaviour in a fixed-duration motion discrimination task, the model integrates sensory evidence transiently, giving rise to a decaying bottom-up CP component. However, the dynamics of the hierarchical loop recruits a concurrently rising top-down component, resulting in sustained CP. We compute the CP time-course of neurons in the medial temporal area (MT) and find an early transient component and a separate late contribution reflecting decision build-up. The stability of individual CPs and the dynamics of noise correlations further support this decomposition. Our model provides a unified understanding of the circuit dynamics linking neural and behavioural variability.

## Abstract (web summary)

The activity of sensory neurons can be correlated with perceptual decisions and this effect may provide insights into how sensory information is processed during perceptual tasks. Here the authors develop a network model of sensory and decision-making areas and propose that the dynamics across the network hierarchy explains the choice probabilities.

## Graphical abstract

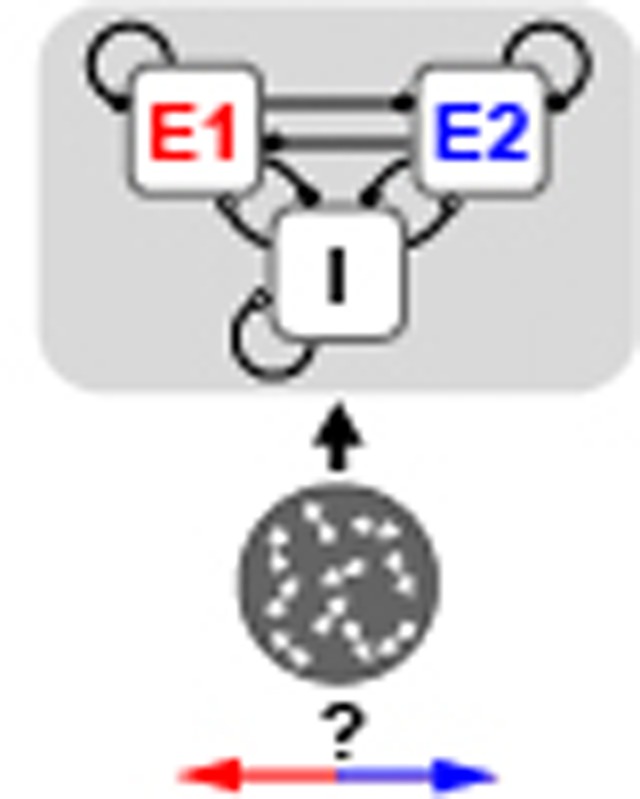

The neural basis of decision making has been studied extensively in monkeys performing perceptual discrimination tasks^1^. One influential finding is that the response variability of single neurons in visual areas such as MT is predictive of the monkey’s choice. A common measure of this correlation is ‘choice probability’ (CP)^2^, the probability that an ideal observer can predict the monkey’s choice solely based on the number of spikes fired by a neuron. CPs above chance level have been found consistently across the visual system^3^,^4^, in a variety of discrimination tasks^2^,^5^,^6^,^7^,^8^,^9^,^10^.

Two different interpretations of CP in sensory neurons have emerged: in the bottom-up interpretation, variability in the choice is partly caused by variability in the response of sensory neurons, and CP quantifies this causal relationship^2^. This interpretation can be formalized in a feedforward network model^11^, where (1) the choice is determined by comparing the pooled activity of noisy sensory neurons across two populations with opposite stimulus preferences, and (2) neuronal variability within these populations is positively correlated^11^,^12^. These noise correlations have generally been observed experimentally^10^,^13^,^14^, but their magnitude and spatio-temporal structure seem to vary across areas, species and experimental conditions. In the top-down interpretation^9^,^15^,^16^,^17^, the variability of sensory neurons that correlates with choice arises due to trial-to-trial fluctuations in top-down signals, which modulate the magnitude of the evoked responses^18^,^19^,^20^. The nature of these top-down signals remains, however, largely unknown: it is not clear on what time-scale they operate^16^, what causes their variability, and whether they are generated before the stimulus presentation, reflecting some kind of bias or expectation, or they are instead recruited by sensory inputs as some kind of bottom-up attentional signal. In any case, CP due to top-down inputs reflects computations that escape the control of the experimenter and cause trial-to-trial response variability that is not necessarily noise.

To differentiate between bottom-up and top-down mechanisms, a recent study compared the dynamics of sensory evidence integration and the time-course of CP in a disparity discrimination task^9^. They found that the impact of stimulus fluctuations on the decision decreases over time, whereas CP increases and reaches a plateau. This indicates that CP cannot be exclusively due to the causal effects of sensory activity on the decision and supports a non-causal relation through top-down signals. However, top-down connections from associative to sensory areas could give rise to recurrent loops across the cortical hierarchy, questioning the rationale of establishing the direction of causality. Whether this recurrent interaction exists and how it may impact the dynamics of sensory integration remains to be elucidated. A further challenge for interpreting CP is that it is directly linked to the structure of noise correlations^12^, but the sources of correlations are not well understood. On one hand, it has become clear that correlations are not a fixed hard-wired property of sensory circuits but depend on a number of factors including the context of the task^14^ and attentional states^18^,^21^,^22^. On the other hand, theoretical work has shown that shared inputs do not necessarily cause correlations in recurrently connected networks^23^, so that we currently lack a canonical network model that can generate a structure of noise correlations as measured experimentally. The emerging view is that correlations do not have a unique origin but can be caused, in addition to hard-wired connectivity, by feedforward (for example, eye movements^24^ or stimulus fluctuations^25^), intrinsic (for example, stochastic global fluctuations of ongoing activity^26^) and top-down sources^14^,^20^, making CPs hard to interpret^3^,^4^.

Here, we present a hierarchical network model of spiking neurons, representing a sensory and an associative cortical area and carrying out the discrimination of two stimulus categories. Noise correlations between sensory neurons together with topographical top-down connections give rise to CP that is generally composed of two contributions: a bottom-up component, which peaks after stimulus onset and decreases as the decision is being formed, and a top-down component, which simultaneously increases until reaching a steady level. We analyse single unit and paired unit recordings from a classic motion discrimination experiment^2^,^13^ and show that CPs in MT have an early bottom-up component (<500 ms after stimulus onset) revealed by trial-to-trial stimulus fluctuations. Leveraging on the heterogeneity exhibited by individual CP time-courses, we further show the contribution of a late component, consistent with slowly varying top-down signals that represent the upcoming choice. This slow late component is also revealed by the rising time-course of lagged spike count noise correlations. Thus, our model elucidates how the emergent dynamics, developing across the hierarchy during sensory integration, frames the relationship between neuronal and behavioural variability in perceptual decision tasks.

## Results

### Mechanisms underlying correlations and CP

**Figure 1:**
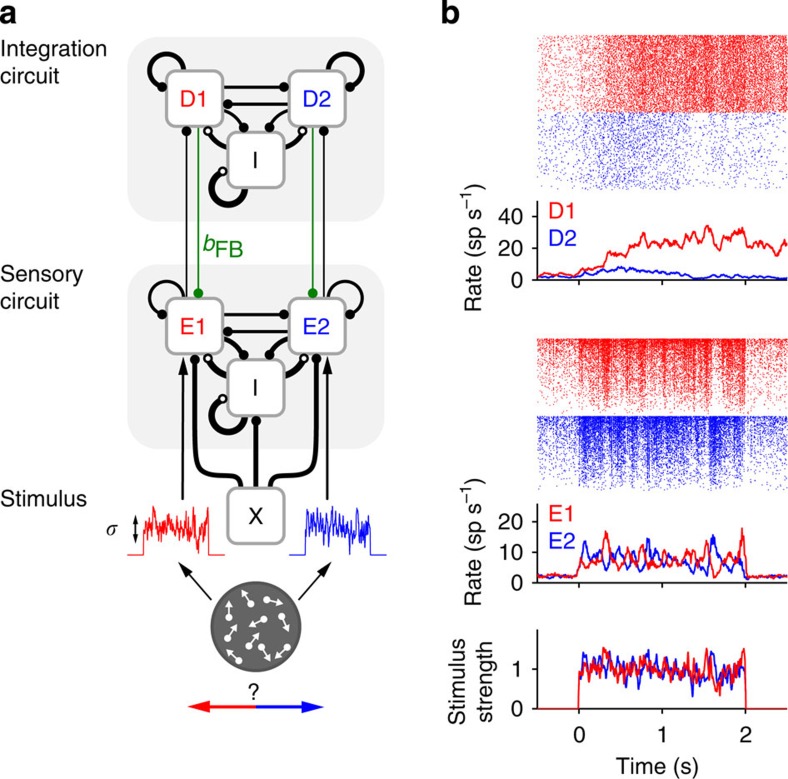
Model architecture and single-trial response. (**a**) Network model composed of a sensory circuit (MT) with two opposite stimulus-selective excitatory populations E1 and E2 that are coupled to two choice-associated populations D1 and D2 in an integration circuit^27^ (for example, LIP, FEF). There are *feedforward* and *top-down feedback* connections (strength *b*_FB_) between the two circuits as well as lateral excitatory and inhibitory (population I) recurrent connections within each circuit (connections are represented by lines with a width proportional to the synaptic efficacy and connection probability). The stimulus is modelled as time-varying input currents to neurons in E1 and E2 (red and blue traces show two examples, s.d. *σ*) mimicking temporal variations in the momentary sensory evidence in favour of one or the opposite direction of motion of an RDK. In addition, sensory neurons receive shared external background Poisson inputs (population X) and decision neurons non-shared Poisson inputs (not shown). (**b**) Response of the network to an example zero-coherence stimulus (*σ*=1). Bottom traces show the population-averaged stimulus currents into E1 and E2. Rastergrams show the spiking activity of neurons in E1 and E2 (middle, 800+800 neurons) and in D1 and D2 (top, 240+240 neurons), sorted by rate. Traces below the rastergrams show the corresponding instantaneous population rates (count window *T*=50 ms). Top-down connections were set to zero (*b*_FB_=0).

**Figure 2:**
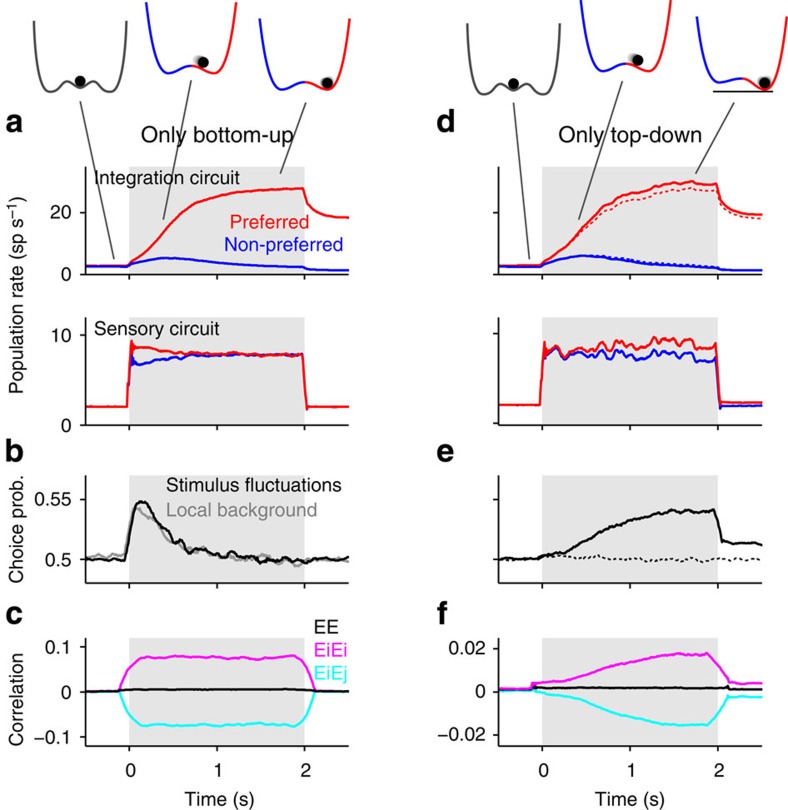
Impact of bottom-up and top-down correlations on choice probability. (**a**–**c**) Network without top-down connections receiving *non-replicate* zero-coherence stimuli that cause bottom-up correlations (see Methods and Supplementary Fig. 1; *b*_FB_=0, *σ*=1). Average population rates for trials yielding the preferred (red) and the non-preferred choice (blue) for the integration and sensory circuits (**a**). Time-courses of the average CP (**b**; count window *T*=100 ms) and average spike count noise correlations (**c**; *T*=250 ms) of sensory neurons. Correlations are shown for pairs of excitatory neurons within (EiEi, pink) and across sensory populations (EiEj, cyan), and of all pairs (EE, black). Average CP, obtained when bottom-up correlations are generated by local background inputs and replicate stimuli, is shown for comparison (grey trace in **b**; see Supplementary Figs 2 and 3 for details). *Upper inset:* after stimulus onset, the dynamics of the integration circuit is described by a double-well energy landscape where each minimum corresponds to a choice attractor. When approaching one of the attractors, the impact of sensory activity fluctuations on the state of the integration circuit decreases. (**d**–**f**) Same as **a**–**c** but using a network with top-down connections (*b*_FB_=1, *σ*=1). We eliminated bottom-up correlations by using replicate stimuli and global background inputs (Methods). The rates and CP obtained without top-down connections are shown for comparison (dotted lines in **d** and **e**). *Upper inset:* the stability of the choice attractor is increased (represented by an increase in well depth) due to the bottom-up/top-down loop dynamics. Shaded areas represent the stimulus interval.

We developed a computational model of perceptual decision making that allowed us to isolate and quantify the dynamics and the relative contributions of bottom-up and top-down mechanisms to spike count noise correlations and CP of sensory neurons. We simulated a standard two-alternative forced-choice motion discrimination task using fixed-duration random dot kinematograms (RDKs)^1^,^2^,^3^,^13^. The spiking network consists of an integration circuit (for example, LIP, FEF) and a sensory circuit (MT), recurrently coupled via bottom-up feedforward connections and top-down feedback connections (Fig. 1a). The integration circuit accumulates sensory evidence and produces a binary categorization due to winner-take-all competition between two decision-encoding populations^27^. The sensory circuit contains neural populations selective to opposite directions of motion, the average responses of which vary approximately linearly with the stimulus coherence. We primarily studied responses to ambiguous, zero-coherence stimuli, which maximize behavioural variability. We used stimuli that caused strong temporal modulation of sensory population rates (Fig. 1b) comparable to those produced by the temporal variations in motion energy caused by RDKs in MT neurons^28^,^29^ (see Methods). These time-varying rates are integrated by populations D1 and D2 in the integration circuit until the network reaches the attractor state associated with one of the choices^27^ (Figs 1b and 2a). Because we are interested in the relationship between neuronal and choice variability, we set the sensory circuit to operate in the balanced regime^30^,^31^ where neurons exhibit large response variability^32^. We wanted to characterize the structure of noise correlations, as it provides the link between single neuron variability and behaviour^11^,^12^. We classified correlations among sensory neurons as bottom-up or top-down, depending on whether they were generated in the absence or by virtue of top-down feedback connections, respectively.

To study the dynamics of CP caused by bottom-up correlations only, top-down connections in the network were removed. We first noticed that because the sensory circuit operated in the balanced regime, average correlations were marginally small despite the presence of anatomically shared inputs^23^. As a consequence, the network did not give rise to substantial CP. We therefore investigated two other potential sources of bottom-up noise correlations in the model: (1) trial-to-trial stimulus fluctuations and (2) the spatial arrangement of external background inputs to the sensory populations. To generate trial-to-trial stimulus fluctuations, we used different realizations of the time-varying zero-coherent stimulus in each trial. This condition, generally used in experiments, was termed non-replicate to distinguish it from repeated presentations of identical replicate stimuli. As a consequence of the non-replicate condition, neuronal pairs within the same population (E1E1 and E2E2) showed positive trial-to-trial correlations (Fig. 2c) reflecting the co-modulation of the evoked rates around the mean response obtained over different zero-coherent stimuli (Supplementary Fig. 1). Thus, these correlations should technically be termed signal correlations instead of noise correlations. Across-population correlations (E1E2) were negative due to competition between E1 and E2 mediated by common inhibition (Supplementary Fig. 2 and Methods). Thus, the difference between correlations within the same population and correlations across populations was large and constant throughout the stimulus period (Fig. 2c), a necessary condition to yield sustained CP^12^. Despite this, CP showed a fast rise followed by a slow decay towards chance level (Fig. 2b). This CP time-course is a direct consequence of the non-linear dynamics of the integration circuit, which, as the trial progresses, approaches an attractor causing a decrease of the impact of the sensory activity fluctuations on the upcoming decision^27^,^33^ (Fig. 2a inset). We call this effect transient evidence integration.

The same qualitative structure of bottom-up correlations was generated by, instead of using non-replicate stimuli, modifying the spatial arrangement of the external background inputs. In circuits with strong global inhibition, spatially localized external inputs are more effective in generating large amplitude fluctuations in population rates than non-specific global background inputs^34^ (Supplementary Fig. 2). Thus, local background inputs specifically targeting E1 and E2 were able to generate correlations with the same magnitude and structure as stimulus fluctuations, leading to virtually identical CP time-courses (Fig. 2b). In addition, the correlations produced by local background inputs caused a substantial decrease in the accuracy of the categorization^11^,^35^ (Supplementary Fig. 3d), which was already suboptimal in the absence of correlations due to transient evidence integration^27^ (Supplementary Fig. 3e).

Given the generality of transient integration, the same qualitative CP time-course is obtained across a broad range of correlation amplitudes (Supplementary Fig. 4), if correlations are caused by other bottom-up sources (for example, inherited from an upstream area), or if the integration circuit is replaced with a bounded integrator^29^ (Supplementary Fig. 5b). Thus, given the invariance of the CP dynamics to the exact origin of bottom-up correlations, we used stimulus fluctuations to develop our experimental predictions as they are easy to manipulate.

Next, we investigated the impact of top-down signals by including weak feedback connections from the neurons in the integration circuit to the corresponding sensory populations (D1 to E1, D2 to E2; Fig. 1a). To isolate the effect of top-down feedback, we removed bottom-up correlations by using global background inputs and by presenting replicate stimuli. As evidence supporting one choice built up in the integration circuit, top-down inputs projected this gradual increase in activity and produced a small boost in the rate of the corresponding sensory neurons, particularly towards the end of the stimulus period (Fig. 2d). This choice-dependent increase in rate generated both correlations between sensory neurons and a CP with a ramp-and-plateau time-course mirroring the build-up of the decision (Fig. 2e,f). The amplitude of the CP increased with the strength of top-down connections (Supplementary Fig. 4e–g).

**Figure 3:**
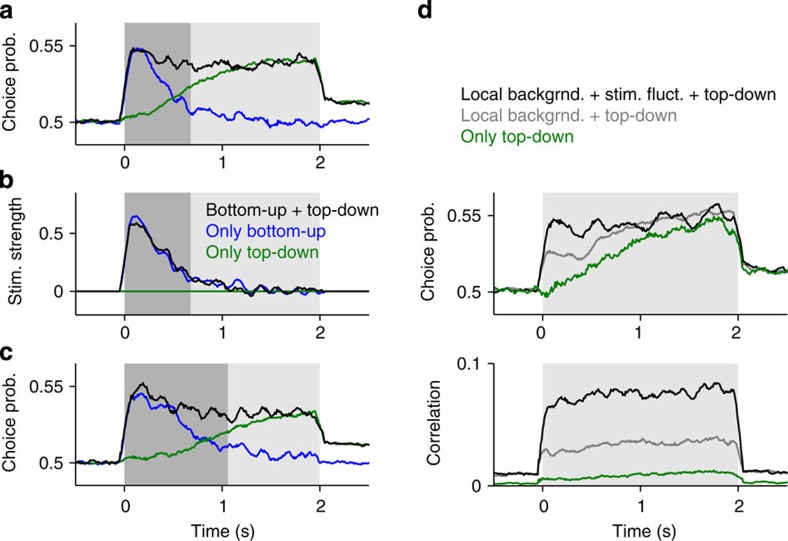
Bottom-up correlations together with top-down signals can lead to sustained CP and a decaying psychophysical kernel. (**a**) CP obtained in the network with top-down connections receiving non-replicate stimuli (*σ*=1, *b*_FB_=1; black) is sustained throughout the stimulus interval (shading). Two complementary contributions to CP are revealed by using replicate stimuli, thus removing bottom-up correlations (green) or by removing the top-down connections (*b*_FB_=0; blue). (**b**) Psychophysical kernel for the three cases illustrated in **a**. The ‘integration window’ (dark shading) is defined as the interval containing 85% of the kernel’s total area. (**c**) Same as **a**, for a network with slower decision dynamics (Methods). Despite the longer integration window (dark shading), the CP of the combined condition remains approximately invariant (black). (**d**) CP and correlations in a network with top-down connections in which bottom-up correlations are produced by both trial-to-trial stimulus fluctuations and local background inputs (see Supplementary Figs 2 and 3). Using replicate stimuli (grey trace) removes part of the bottom-up contribution to CP and correlations. In addition, making the background inputs global isolates the contribution of top-down connections (green). Count windows were *T*=100 ms.

When taken in isolation, neither bottom-up correlations nor top-down connections could account for the fast rise in CP followed by a plateau that is observed experimentally^2^. However, when taken together, the two mechanisms could reproduce this time-course (Fig. 3a). The resulting CP was approximately the sum of the CPs obtained due to either bottom-up or top-down contributions alone, regardless of whether bottom-up correlations were caused by one or several factors (Fig. 3d). The sustained CP time-course was not a general feature of the model but depended on the relative strength of these contributions (Supplementary Figs 3b and 4a,e). However, the decaying bottom-up and the rising top-down component were both governed by the dynamics of sensory evidence integration and thus evolved with the same time-scale. This made CP roughly invariant to changes in decision dynamics (Fig. 3a,c). The network’s psychophysical kernel, obtained from the difference between the average stimuli yielding each choice^9^,^29^ (Fig. 3b), revealed that, despite the sustained CP, the network performed transient integration of sensory evidence. Finally, the decomposition of CP into bottom-up and top-down components was also qualitatively unchanged if the average correlation across all pairs, which was close to zero in our model (Fig. 2c,f), was increased while maintaining the difference corr(EiEi)—corr(EiEj) (Supplementary Fig. 6).

### CP in MT during a motion discrimination task

We then asked whether CP in MT neurons during a fixed-duration motion discrimination task could also be decomposed, like in the model, into (1) an early, bottom-up component, reflecting in part the impact of stimulus fluctuations and (2) a late, top-down component that reflects feedback from an integration circuit. We reanalysed responses to zero-coherence RDKs from classical monkey experiments^2^,^13^, which yield a sustained time-course of the population-averaged CP^2^ (Supplementary Fig. 7a). However, sustained CP could also be explained by perfect evidence integration throughout the whole stimulus period (that is, without a bound) in the absence of top-down signals (Supplementary Fig. 5e). This is unlikely because perfect integration weighs the evidence uniformly, yielding a sustained psychophysical kernel (Supplementary Fig. 5f), in contrast to the decaying psychophysical kernels obtained in similar tasks^9^,^29^ and in two monkeys that we trained to perform the fixed-duration motion discrimination task^2^,^13^ (Supplementary Fig. 7c,e; Methods).

**Figure 4:**
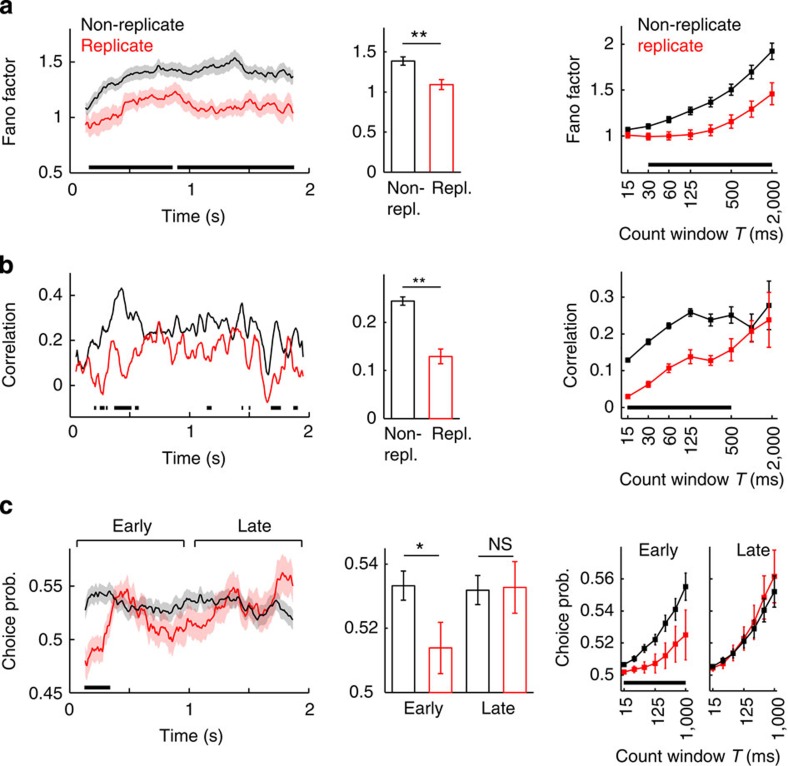
Stimulus fluctuations impact neuronal variability and reveal a bottom-up component of choice probability in MT. Left panels show the time-course of the spike count Fano factor (**a**), spike count correlation (**b**) and CP (**c**) obtained from responses to non-replicate and replicate stimuli using adjusted count windows *T*=250 ms (**a**,**c**) and 100 ms (**b**). The duration of the sliding count window was adjusted to equalize the mean spike count in all time points (Methods). Fano factor and CP are population averages using only zero-coherence trials (replicate: *n*=41 neurons and 41 different RDKs; non-replicate: *n*=118 neurons and 7,733 different RDKs). Correlation was obtained for a single neuronal pair^25^ (emu035; Supplementary Fig. 8b shows the correlation of a second pair), averaging over coherences (range: 0–51.2%, Methods). Centre panels show time-averages obtained from the entire stimulus interval for the Fano factor and correlations (*P*<0.001; permutation tests), and separately for early (0-1 s; *P*=0.02, permutation test) and late epochs (1-2 s; *P*=0.63, permutation test) for the CP. Right panels show the dependence on the window size, using non-overlapping windows of fixed duration *T*. Error bars indicate s.e.m. Thick horizontal lines mark periods of significant difference between the non-replicate and the replicate conditions (*P*<0.05, permutation test).

We first searched for evidence in the MT data of an early bottom-up component of CP that could be revealed by manipulating the magnitude of bottom-up sensory correlations. To do this, we compared responses in the non-replicate and the replicate conditions, as a way to assess the impact of bottom-up correlations, caused by trial-to-trial stimulus fluctuations, on CP^2^,^10^,^25^,^28^,^36^. We first confirmed that stimulus fluctuations had a sustained impact on neuronal variability, as we found substantially lower spike count Fano factors and correlations in MT for the duration of the stimulus in the replicate condition^25^ (Fig. 4a,b). We then tested the prediction of our model, that replicate stimuli should produce CP of smaller magnitude, particularly early during the stimulus presentation, while the network is integrating the sensory evidence, and not late, when CP is mainly reflecting the impact of top-down inputs (Fig. 3a,d). Indeed, we found that CP in the replicate condition was significantly lower than for the non-replicate condition early (epoch 0–1,000 ms), but not late (epoch 1,000–2,000 ms), in the trial (Fig. 4c). Significance was assessed using a mixed-effects ANOVA (with factors epoch (early/late), stimulus (repl./non-repl.), and random factors monkey and neuron identity) for 250 ms, showing a significant interaction effect of epoch × stimulus, F_(1,317)_=4.75, *P*=0.03, *n*=41 (118) neurons for repl. (non-repl.). One-tailed *t*-tests revealed a significant difference of repl. versus non-repl. for early (CP(repl.)=0.512±0.008, CP(non-repl.)=0.532±0.005, *P*=0.02, *t*(157)=−2.07) but not late (CP(repl.)=0.533±0.011, CP(non-repl.)=0.529±0.006, *P*=0.72, *t*(157)=0.353). Thus, a substantial part of CP early in the trial can be attributed to bottom-up correlations caused by stimulus fluctuations. The higher impact of bottom-up correlations early in the trial is a signature of transient integration of sensory evidence and rules out the possibility that the monkeys performed perfect integration (Supplementary Fig. 5e,f). The similar CP late in the trial is consistent with the proposed top-down component, which is present independently of the stimulus condition (replicate versus non-replicate).

### Stability of individual CPs increases through the trial

**Figure 5:**
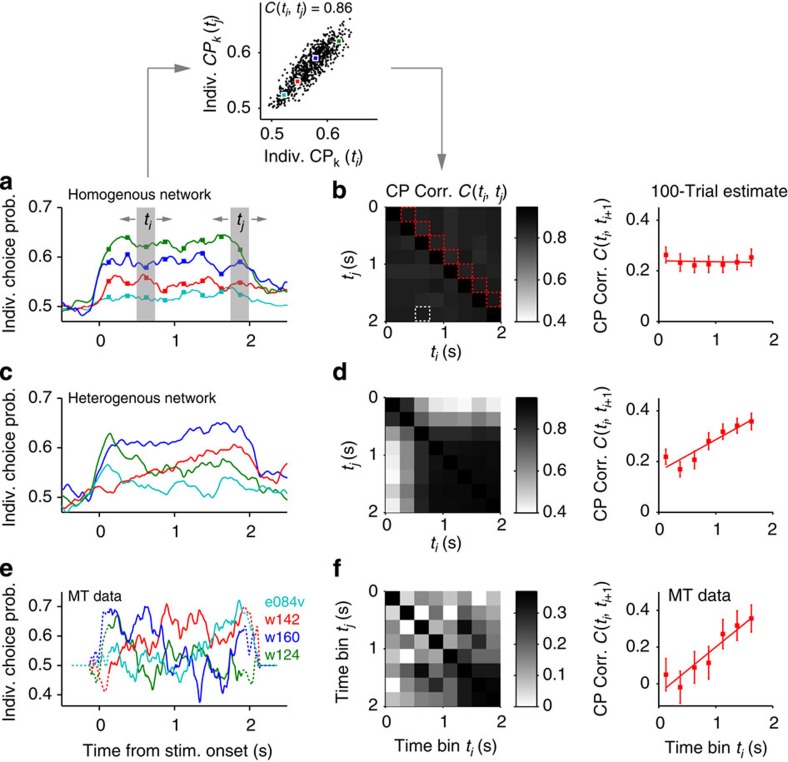
Heterogeneity of individual choice probabilities reveals increased stability towards the end of the stimulus interval. (**a**,**b**) Individual CP traces of four example cells (**a**) and CP correlation matrix *C(t*_*i*_, *t*_*j*_) (**b**) obtained from the homogeneous network (same network as used in Figs 1, 2, 3; CP estimated from 2,000 trials, count window *T*=250 ms). Top inset: each dot is the individual CP of one neuron computed in two time bins *t*_*i*_ and *t*_*j*_ and *C(t*_*i*_, *t*_*j*_) is the rank correlation coefficient across all neurons (Methods). The values shown correspond to the time bins shaded in **a** and marked with a white square in **b**. Coloured dots correspond to the four cells shown in **a**. (**b**) CP correlation matrix *C(t*_*i*_, *t*_*j*_) shows uniformly high values capturing the maintenance of the sorting of individual CPs across time. Right: Time-course of adjacent CP correlations *C(t*_*i*_, *t*_*i+1*_) (red diagonal in **b**) where CPs were estimated from 100 trials to compare with data. The solid line shows a linear fit. Increased number of trials increased the correlation values but did not change the results qualitatively (Supplementary Fig. 9). (**c**,**d**) Same as **a**,**b**, for the heterogeneous network (Methods). The individual CP traces are from representative neurons belonging to four different groups depending on whether they receive stimulus and/or top-down inputs (see Methods and Supplementary Fig. 9). The matrix *C(t*_*i*_, *t*_*j*_) shows an elevated plateau towards stimulus offset reflecting late increased stability in individual CPs. (**e**,**f**) Same as **a**,**b**, for the MT data^2^,^13^ (*n*=143 neurons; variable trial numbers, range: 25–221, median: 59) recorded in the non-replicate condition (see Supplementary Fig. 9 for replicate condition). Error bars indicate the s.e.m. Dotted lines in **e** indicate windows straddling stimulus onset or offset.

We next searched for evidence of a separate contribution to CP caused by late top-down inputs by investigating the heterogeneity of CP profiles across MT neurons. We reasoned that if CP is generated by two separate mechanisms, individual neurons might not participate equally in the two, particularly if they come from layers with a different density of bottom-up versus top-down inputs. So far, we used a homogeneous network, in which all sensory neurons received a stimulus input with the same strength, and top-down connections with equal probability. In the homogeneous network, when the average CP was sustained, individual CP time-courses caused by non-replicate stimuli were also sustained (Fig. 5a), only showing heterogeneity in their amplitude due to heterogeneous firing rates. This stability of CPs over time can be captured by the rank correlation across neurons of the CP measured at two different time bins (CP correlation *C*(*t*_*i*_*,t*_*j*_); Fig. 5a inset), which remains high for all time bin pairs (Fig. 5b). In particular, the CP correlation between adjacent time bins *C*(*t*_*i*_*,t*_*i+1*_) was constant through the trial (Fig. 5b, right). Introducing heterogeneity in the efficacies of stimulus and top-down inputs in the circuit (Methods) can yield sustained average CP and heterogeneous individual CP time-courses (Fig. 5c and Supplementary Fig. 9). Individual profiles showed combinations of fast-rise-and-decay and slow-ramp-and-plateau behaviour, depending on whether they received stimulus and/or top-down inputs (Fig. 5c). Consequently, CPs between adjacent time bins were less correlated at the beginning of the trial (Fig. 5d) because the impact of trial-to-trial stimulus fluctuations made the individual CPs change rapidly (Fig. 3a). Correlations between adjacent bins increased towards the second half of the trial as individual CPs became more stable (Fig. 5d). This analysis is robust to small trial number (Supplementary Fig. 9).

Individual CP time-courses of MT neurons in response to non-replicate stimuli also showed large heterogeneity (Fig. 5e) in spite of yielding a sustained average CP (Supplementary Fig. 7a). As in the heterogeneous network, CP correlations between adjacent time bins were low at the beginning and increased significantly through the trial (Fig. 5f; linear regression intercept=–0.05 and slope=0.25 s^–1^ significantly different from zero, *P*<0.001, permutation test). Moreover, the average CP correlation across time bin pairs within the first half of the trial was significantly smaller than within the second half (mean of *C*(*t*_*i*_*,t*_*j*_)=0.096±0.026 for *t*_*i*_, *t*_*j*_<1 s, *i*≠*j*, and 0.275±0.025 for *t*_*i*_, *t*_*j*_>1 s, *i*≠*j*, respectively; *P*<0.001, *n*=6 time bin pairs, permutation test). Thus, individual CP time-courses in MT were heterogeneous and showed increasing stability towards the end of the stimulus, consistent with the slow build-up of a top-down contribution.

### Lagged correlations predict late CP

**Figure 6:**
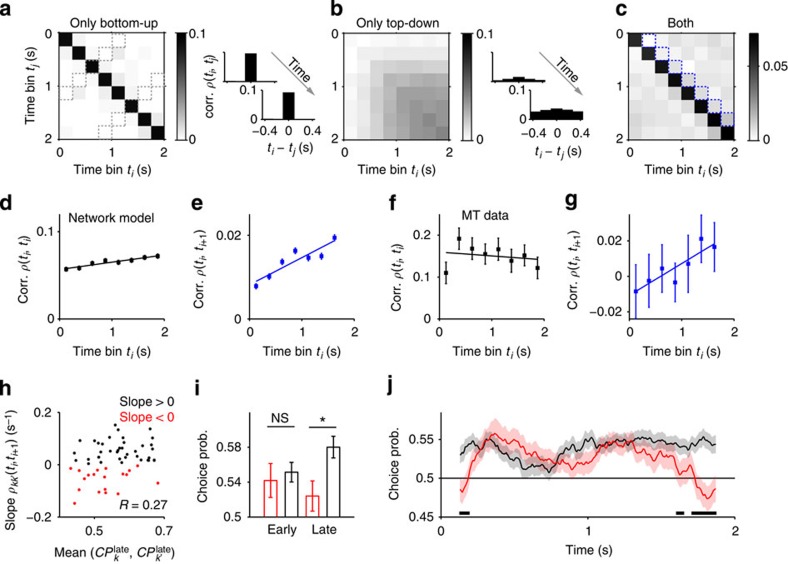
MT neuron pairs with rising lagged correlations exhibit enhanced late CP. (**a**–**c**) Spike count correlations obtained from neuron pairs in the homogeneous network with bottom-up correlations but no top-down inputs (**a**), with top-down inputs and no bottom-up correlations (**b**), and in the heterogeneous network with both top-down inputs and bottom-up correlations (**c**). The matrix entries *ρ(t*_*i*_, *t*_*j*_) are correlation coefficients of the spike counts in time bins *t*_*i*_ and *t*_*j*_ (count window *T*=250 ms) averaged over cell pairs. *Insets*: correlations *ρ(t*_*i*_, *t*_*j*_) versus time lag *t*_*i*_*−t*_*j*_ from two instants of the stimulus interval (dashed diagonals in **a**). Bottom-up correlations were generated by non-replicate stimuli. (**d**,**e**) Time-course of correlations in the full model (**c**) shows large amplitude sustained instantaneous correlations (**d**; main diagonal in **c**), caused mainly by stimulus fluctuations (see **a**), and slowly rising lagged correlations (**e**, blue dashed diagonal in **c**), caused by top-down inputs (see **b**). (**f**,**g**) MT correlations (*n*=32 neuron pairs at coherences −3.2, 0, +3.2%) show similar time-courses as the model: instantaneous correlations (**f**) do not change significantly over time whereas lagged correlations (**g**) increase significantly (regression line slopes −0.0092, s^−1^ and 0.018 s^−1^, with *P*=0.718 and *P*=0.046, respectively; permutation tests). The weak non-monotonic trend of the instantaneous correlations (fast-rise + slow-decay) shown in **f** can be partly due to the similar trend displayed by the evoked rate^69^ (not shown). (**h**) Slopes of lagged correlations *ρ*_*kk'*_*(t*_*i*_, *t*_*i+1*_) for individual MT pairs versus the mean late CP of the two corresponding neurons (correlation *R*=0.27, *P*=0.02). (**i**) Average early and late CP for pairs with positive (red) and negative (black) slopes of lagged correlations (early: 0–1 s, late: 1–2 s; *T*=1,000 ms). (**j**) Average CP time-course for cell pairs showing rising (black) and decaying (red) lagged correlations (*T*=250 ms). Error bars indicate s.e.m. Thick horizontal lines mark periods of significant difference (*P*<0.05, permutation test).

Finally, we looked for further evidence of a late and slow top-down contribution to CP by analysing spike count correlations in MT simultaneous pair recordings^13^,^25^. In the model, pairwise correlations produced by stimulus fluctuations were short-lived (~50–100 ms; Fig. 6a), mimicking fluctuations in motion energy at the speed of the coherent motion produced by RDKs (Methods). In contrast, correlations caused by top-down inputs were weaker, but as they built up (Fig. 2f), they extended to time lags of a few hundred milliseconds (Fig. 6b). This is because these correlations are caused by trial-to-trial variations in the population rates of the integration circuit that remain relatively stable during the late part of the stimulus (Fig. 1b). When both sources are combined (Fig. 6c), the model predicts large and sustained correlations at zero lag (Fig. 6d) and weak lagged correlations with a rising time-course (Fig. 6e). We tested this prediction in MT pairs and found that both the instantaneous and lagged correlations averaged over pairs resembled those predicted by the model (Fig. 6f,g). Moreover, individual pairs with rising lagged correlations tended to exhibit large values of late CP (Fig. 6h). This is consistent with our heterogeneous model, where rising lagged correlations and increasing CP time-courses were specific of neural pairs receiving top-down inputs (Supplementary Fig. 10). We thus divided MT pairs in two groups based on the sign of the slope of lagged correlations (Fig. 6h). As predicted by the model, neurons showing rising lagged correlations yielded a larger late CP than neurons showing a decay (Fig. 6i,j). The difference in CP specifically occurred late in the trial (Fig. 6i,j; mixed-effects ANOVA with factors epoch (early/late), slope (positive/negative), and random factor neuron identity, revealed a significant interaction epoch × slope, F_(1,129)_=4.14, *P*=0.046, *n*=22 (43) for positive (negative) slope, and a significant difference of negative versus positive for late but not early, *P*=0.007, *t*(63)=2.55 and *P*=0.33, *t*(63)=0.443, one-tailed *t*-tests). Neurons with rising lagged correlations also showed higher CP at stimulus onset. We speculate that this difference could be due to pre-stimulus expectation signals^9^, not implemented in the model, that modulate sensory activity using the same top-down pathway.

### Top-down feedback enhances the stability of the categorization

**Figure 7:**
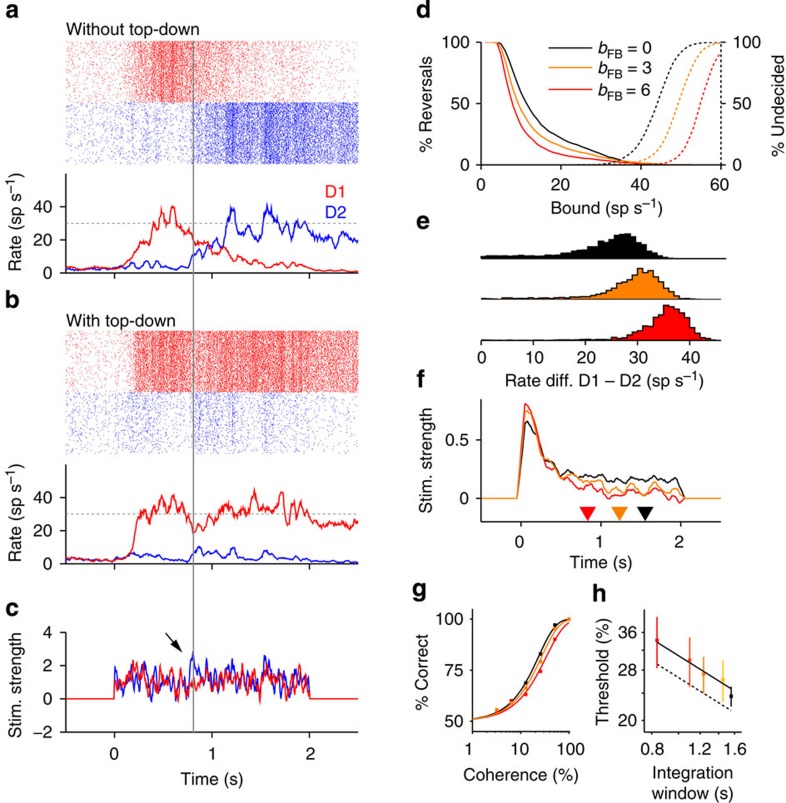
Top-down inputs strengthen the categorization dynamics and increase their stability. (**a**,**b**) Spike rastergram and population rates of the decision populations D1 and D2 (240+240 neurons) during an example trial for the network without (**a**) and with (**b**) top-down connections (*b*_FB_=0 and *b*_FB_=4, respectively). The same zero-coherence stimulus (**c**) with large temporal modulation (*σ*=2.7) caused population D1 to initially cross the arbitrary categorical bound in both cases (dotted line). A large fluctuation (arrow) reversed the state in the network without top-down (**a**, reversal trial) but not in the network with top-down connections (**b**). (**c**) Population-averaged stimulus currents into sensory E1 neurons (red) and E2 neurons (blue). (**d**) Percentage of reversal trials (solid) and no-bound-crossing trials (dotted) versus value of the non-absorbing bound for different top-down strengths (see inset). (**e**) Distribution of the population rate difference D1−D2 averaged over the second half of the stimulus interval (1-2 s) for trials in which the choice was ‘1’. Stronger top-down inputs increased the differences in rate and led to fewer trials with rate difference close to zero (*weak-confidence* trials). (**f**) Psychophysical kernel shows that the integration window (interval containing 85% of the total area under the kernel; triangles) shortens with increasing top-down strength. (**g**) Percentage of correct choices as a function of stimulus coherence. Simulation data (squares) were fitted using a Weibull function (solid lines). (**h**) As the top-down strength decreases, the discrimination threshold, defined as the coherence yielding 82% of correct trials, decreases as one over the square root of the integration window length (solid line is a fit of slope of −0.5 in the log-log graph). The network performance is only slightly worse than the perfect integration of evidence during the integration window (dotted line). Different colours represent top-down strengths (*b*_*FB*_=0, 1.5, 3, 4.5 and 6).

Functionally, top-down connections generated a positive feedback loop across the hierarchy that modified the decision dynamics and enhanced the stability of the categorization (Fig. 7). We arbitrarily defined a non-absorbing bound in the firing rate that, when reached by population D1 or D2, indicates strong evidence in favour of motion in the corresponding direction. After the first bound crossing, the attractor dynamics of the integration circuit tended to maintain this state until the end of the stimulus when the decision had to be taken^27^. This occurred unless a large fluctuation in sensory activity reversed the competition between D1 and D2 (ref. 27) (Fig. 7a). Adding top-down connections altered the dynamics by preventing some reversals observed in the network without top-down (Fig. 7b). This occurs because, when approaching the bound, top-down feedback generates a difference between the rates of the two sensory populations (Fig. 2d) that enhances the stability of the current state of the integration circuit (Fig. 7b and Fig. 2d inset). Thus, stronger top-down feedback yielded fewer reversals (Fig. 7d). Raising the bound decreased the fraction of reversal trials but increased the fraction of trials without threshold crossing (that is, weak-confidence trials^37^). Stronger top-down feedback increased the difference between D1 and D2 rates at stimulus offset, resulting in fewer weak-confidence trials (Fig. 7d,e). Consistent with fewer reversals (Fig. 7d), stronger top-down feedback yielded shorter integration windows and a weaker impact of late stimulus fluctuations on the decision (Fig. 7f). Enhanced stability came at the cost of decreased discrimination accuracy across a broad range of stimulus coherences^27^ revealing a trade-off between stability and accuracy (Fig. 7g). This trade-off can be portrayed as the standard speed-accuracy trade-off: shorter integration windows, resulting from stronger top-down inputs, yielded higher discrimination thresholds (Fig. 7h). Comparing the network’s performance with an optimal classifier shows that the decrease in accuracy due to the increase in top-down strength can be entirely attributed to the concurrent shortening of the integration window (Fig. 7h). Thus, the strength of top-down feed-back could be optimized to maximize reward rate depending on task details such as stimulus duration and the cost of erroneous and undecided trials.

## Discussion

We have developed a hierarchical network model to investigate how CP, the correlation between neuron response variability and perceptual decisions, emerges from recurrent cortical network dynamics. The model dissects CP into an early contribution, dominated by the impact of bottom-up trial-to-trial neuronal co-fluctuations on the decision, and a late contribution produced by top-down inputs into sensory neurons, which reflects the decision build-up in each trial. The time-courses of the two contributions are determined by the non-linear integration of sensory activity resulting from the dynamics of the hierarchical network: as the stimulus comes on, sensory evidence is gradually accumulated, until the integration circuit reaches a categorization state that is then maintained by the reverberant activity of the local circuit^27^ as well as across the hierarchy. As this categorization state is much less sensitive to sensory fluctuations, bottom-up contributions to CP are confined to the initial accumulation phase and decay over time. Top-down contributions show a complementary rising time-course because they reflect the build-up and maintenance of the decision. When the two contributions have comparable magnitude, CP can exhibit a sustained time-course for a wide range of decision dynamics as observed in the classical motion discrimination task^2^. The model, however, predicts that if the magnitude of the two contributions is different, CP should show a non-sustained time-course. We implemented the integration circuit using a spiking attractor network model^27^. However, the decomposition of CP into complementary bottom-up and top-down contributions can be obtained with a broad family of decision models, including a firing rate attractor network model^33^,^38^ or a linear integrator with absorbing bounds in combination with a post-decision feedback signal, but not with a leaky^29^ or a perfectly linear integrator (no bounds).

We used trial-to-trial stimulus fluctuations (‘non-replicate’ condition) as one way to generate bottom-up correlations, that is, those generated in the absence of top-down connections, and to establish predictions that could be easily tested experimentally. While previous studies concluded that the impact of stimulus fluctuations on CP and neuronal variability was small or negligible^10^,^25^,^36^,^39^, we found that non-replicate RDKs caused a substantial increase in spike count Fano factor and correlations compared with replicate RDKs (they accounted for ~35% of the correlations for coherences in the range 0–50% in two pairs of similarly tuned cells, *T*=100 ms). The differences with previous reports could be due to our smaller spike count windows^25^,^36^ and lower coherences^39^. We then compared CPs in MT for replicate and non-replicate stimuli and verified a key model prediction: while replicate stimuli caused a sustained decrease in correlations compared with non-replicate, they generated a decrease of CP only early and not late in the trial.

Strong local excitatory connections in combination with strong and global inhibition in the sensory circuit gave rise to competitive dynamics between the populations E1 and E2. These local dynamics conferred the spike count correlations caused by non-replicate stimuli or by local background inputs a distinctive structure: pairs within one population (EiEi) were positively correlated while mixed pairs (EiEj) were negatively correlated, yielding a near zero average across all pairs. Although similarly tuned MT neurons commonly exhibit larger correlations than oppositely tuned ones^13^,^14^,^39^, only the correlations found in the medial superior temporal cortex (MST) of trained monkeys resemble the structure produced by the model, with negative correlations among pairs with opposed tuning and near zero average correlations across all pairs.^40^ Because in our network CP depends on the difference in average correlations within (EiEi) and across populations (EiEj)^12^, the decomposition into bottom-up and top-down components is invariant to changes in the correlation structure that maintain this difference (for example, an increase in the average correlation across all pairs). Bottom-up correlations caused by other types of stochastic network dynamics such as switches between multiple discrete attractors^41^,^42^ or diffusion in a continuous line attractor^43^,^44^,^45^ would in general also contribute to the early component of CP. Bottom-up correlations with a very slow time-scale produced by intrinsic network dynamics would generate CP that rises and decays slowly, and they could eventually cause above chance CP before stimulus onset^46^. In sum, even if our analysis cannot identify the precise origin of bottom-up correlations, it succeeds in showing that any mechanism causing bottom-up fast correlations leads to a similar fast-rise-and-decay CP time-course.

Top-down inputs carrying decision-related or attentional signals also contribute to CP^9^,^17^ and correlations^14^,^20^. We designed a model-driven analysis that, based on the stability of the single cell CP traces, provided evidence for a late top-down contribution to CP. We defined the CP correlation as a measure of the stability of individual CP traces: early CP produced by bottom-up stimulus fluctuations caused temporally varying traces that crossed frequently yielding low CP correlation. Late in the stimulus, when CP is presumably dominated by slower top-down inputs, CP traces were more stable yielding higher CP correlation. Moreover, we found that lagged correlations between MT pairs showed a rising time-course during the stimulus that was predictive of the magnitude of late CP across cell pairs. Together, these findings are consistent with the presence of a top-down signal that, towards the end of each stimulus, selectively boosts the rate of a subset of MT cells aligned with the upcoming choice. Our analysis suggests why previous reports^25^,^39^ might have missed lagged correlations: they are weak, confined to the late part of the stimulus and could be selectively expressed by a subpopulation of neurons receiving top-down inputs, as in attentional studies in V1 (ref. 20).

The impact of noise correlations on how accurately populations of neurons represent sensory information has typically been assessed in settings where tuning curves and correlation structure are treated as independent variables and where the effect of the dynamics of the recurrent circuitry has been mostly unexplored^47^,^48^,^49^. Our approach is different in that our network function is not to accurately represent the stimulus, but rather to perform a binary categorization. This computation is implemented using strongly recurrent connectivity that induces competitive dynamics^27^. Competition is crucial for the categorization in the integration circuit, but it also emerges in the sensory circuit via top-down feedback and fluctuations in the external inputs affecting both the tuning and the correlation structure. In particular, competition induces negative noise correlations across the two oppositely tuned sensory populations, a condition that impairs discrimination accuracy^11^,^50^. Understanding how variability ultimately constrains function will require a systematic characterization of the relation between connectivity, dynamics and correlations in networks designed to carry out specific computations^51^,^52^.

Our analyses support the idea that late CP in MT cells reflects the impact of top-down inputs on sensory evoked responses^8^,^9^,^15^,^17^. Top-down inputs in this context have been interpreted as selective attentional signals^19^ whose allocation varies on multiple time-scales^18^, introducing co-variability in sensory populations^14^,^20^ and biasing perceptual decisions^17^,^18^. The specific nature and dynamics of these top-down inputs remains largely unknown. They include pre-stimulus expectation signals^53^, reflecting the animal’s ‘guess’ about the upcoming stimulus, or attention signals recruited by the sensory activity produced by salient fluctuations at the beginning of the stimulus. Both of these signals bias sensory activity towards one choice before the subject commits to a decision^9^. Alternatively, they could be post-decision signals, reinforcing the representation of the chosen percept^8^,^9^ or reflecting the motor response plan developing slowly during the stimulus interval^54^. A pure post-decision signal could be implemented by replacing the integration circuit in our network with a linear integrator with absorbing bounds that sends a feedback signal to the sensory circuit once the bound is reached. Such a phenomenological model would yield similar bottom-up and a top-down CP components as our model. However, the top-down signals in our model are not purely post-decision but combine previous proposals of pre-decision and post-decision top-down signals^9^. Because the sensory and integration circuits are recurrently coupled and evolve dynamically in parallel, feedback signals are effectively recruited by activity of sensory circuits and impact the final decision by altering the dynamics during both the accumulation and the categorization maintenance periods^55^. As a consequence, the top-down contribution to CP cannot be called ‘non-causal’ as would be the case for a post-decision signal. The differences between our mechanistic model and the phenomenological integration-to-bound plus feedback model are revealed after the first crossing of the categorization bound: while in our model it is easy to investigate the dynamics of changes of mind, caused for instance by stimuli with time-varying coherence^56^ or by external stimulation^57^, the phenomenological model requires further *ad-hoc* assumptions about the probability to escape the absorbing bound^58^ and about when to switch the top-down input.

During fixed-duration motion discrimination task subjects categorize RDKs and settle on one alternative before the end of the stimulus, ignoring late evidence and performing suboptimally^29^,^59^. Perception appears categorical even for low-coherence RDKs in a task that requires taking into account the probabilities of both motion alternatives to perform optimally^60^. A recent study has proposed that transient evidence integration may arise from approximate inference using sequential neural sampling^61^. Attractor dynamics produced by recurrent lateral connections within integration circuits are a plausible mechanistic implementation of perceptual categorization^27^. Recurrent feedback through top-down connections in our network strengthened this categorization dynamics and increased its stability at the cost of decreased accuracy. Although in our setting these two mechanisms seem to differ only quantitatively, the distinctive features of a top-down implementation would be revealed in tasks requiring the binding of several stimulus attributes (for example, motion and disparity), such as in the discrimination of the direction of rotation of a perceptually bistable cylinder^8^. Our hierarchical model pioneers the analysis of recurrent dynamics in bottom-up/top-down loops, a mechanism that may play a crucial role in the perceptual categorization of more complex stimuli.

## Methods

### Network model

The network model (Fig. 1a) consists of a sensory circuit reciprocally connected to an integration circuit. A standardized description of the model and all simulation parameters can be found in Supplementary Tables 1–4 (ref. 62).

The sensory circuit is a balanced randomly connected EI-network^23^,^30^,^31^ (connection probability *p*=0.2) with 1,600 excitatory (E) and 400 inhibitory (I) leaky integrate-and-fire neurons. Model equations and parameter values are mostly taken from ref. 23. Synaptic transmission mimics AMPA and GABA_A_ receptor conductance dynamics^23^ (mean efficacies 
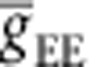
=0.76 nS, 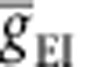
=1.52 nS and 
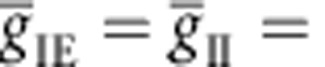
12.6 nS). Excitatory neurons are divided into two symmetric populations, E1 and E2, preferring opposite directions of motion. Connections within each population (E_*i*_ to E_*i*_ with *i*=1, 2) are stronger (potentiating factor *w*_+_=1.3) than connections across populations (E_*i*_ to E_*j*_ with *i*≠*j;* weakening factor *w*_−_=0.7), capturing the stronger coupling among cells with similar direction preference. The stimulus is modelled as a time-varying input current 
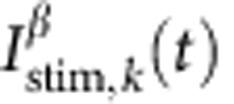
 into each neuron *k* in population *β*=E1 or E2 (see details below). Neurons in E1 and E2 also receive weak top-down connections from populations D1 and D2 of the integration circuit, respectively (Fig. 1a; connection probability *p*_FB_=0.2; synaptic weight *g*_FB_=0.0668, nS·*b*_FB_, with *b*_FB_ the dimensionless feedback strength that takes values in the range 0-6). All sensory neurons receive AMPA-like random connections from a external population (X) composed of 1,000 cells firing independent Poisson spike trains at a constant rate *ν*_ext_=12.5 sp s^−1^ (connection probability *p*_*x*_=0.32; efficacy 
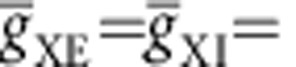
1.71 nS; see Supplementary Fig. 2a for a variant with local background inputs). Spike trains from X cells were always different across trials. Thus, EiEi pairs share on average a fraction of 0.2, 0.32 and 0.2 of their recurrent, external and top-down inputs, respectively.

The integration circuit is a biophysical network model of decision-related activity in LIP^27^, whose dynamics have been extensively studied^33^,^38^. It contains 1,600 excitatory and 400 inhibitory leaky integrate-and-fire neurons, that are all-to-all connected. There are three populations of excitatory cells: D1 and D2 (240 cells each) represent the two choices, and the non-specific population Dn contains the rest of the E cells. Synaptic transmission mimics AMPA, NMDA and GABA_A_ receptor conductance dynamics^27^ (efficacies 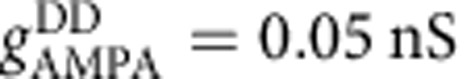, 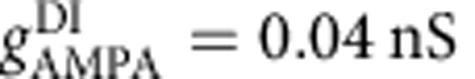, 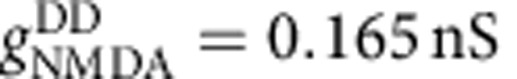, 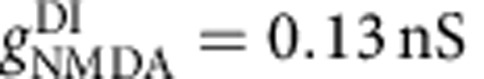, 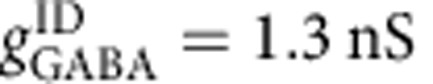, and 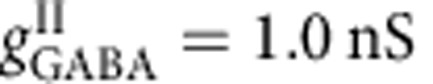). Recurrent connections within D1 and D2 are stronger (factor *w*_+_=1.6) than connections within Dn. Cells in D1 and D2 receive the sensory evidence via feedforward AMPA connections from neurons in E1 and E2, respectively (Fig. 1a; connection probability *p*_FF_=0.2; efficacy 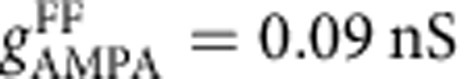). Each neuron in the integration circuit receives an external independent Poisson spike train via AMPA synapses (rate 2,392 sp s^−1^ to D1 and D2 and 2,400 sp s^−1^ to Dn and I; efficacies 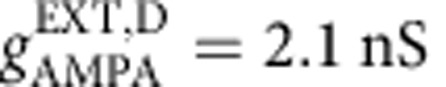, 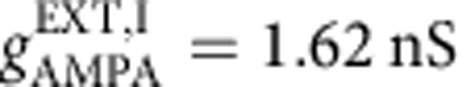). External spike trains were always different across trials.

*Network dynamics*. Although sensory and integration circuits followed the same connectivity scheme (Fig. 1a), the connectivity strengths differed such that they exhibited different dynamics. The sensory circuit generated weak competition between E1 and E2 allowing the network to operate in an approximately linear regime in which each population rate can track the stimulus input (Fig. 1b). This weak competition shaped the structure of correlations^34^ generated by non-replicate stimuli (Fig. 2c), local background inputs (Supplementary Fig. 3c) and top-down signals (Fig. 2f): correlations between pairs within the same population (EiEi) are positive, whereas in mixed pairs (EiEj) they are negative. The average correlation across all pairs was near zero, a robust feature of balanced networks resulting from the dynamic balance of excitation and inhibition^23^. In contrast, on stimulus presentation, the integration circuit exhibited non-linear dynamics as a consequence of strong winner-take-all competition and the existence of two attractors representing the two possible choices^27^,^33^,^38^.

*Stimulus model*. The stimulus-driven input was modelled as an afferent current 
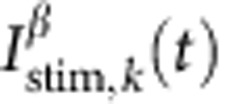
 into each sensory neuron *k* in population *β*=(E1, E2):


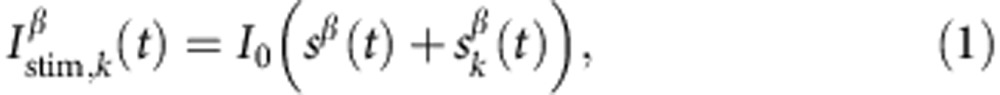


with *I*_0_=0.08 nA, the mean input for zero-coherence stimuli. The term s^*β*^(*t*) is the stimulus, representing sensory evidence for motion in the *β*-direction. It is common to all neurons of population *β* and independent between the two populations (except in Supplementary Fig. 6). The term 
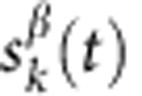
 is independently generated for each neuron *k*, mimicking heterogeneity in the afferent input. Figure 1b, shows an example of *s*^E1^(*t*) and *s*^E2^(*t*) (bottom traces) and Supplementary Fig. 1b,c compares *s*^*β*^(*t*) and 
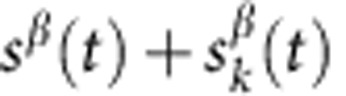
 for two neurons (top traces). The two terms are given by


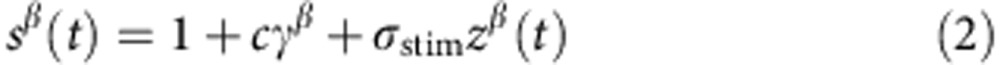


and


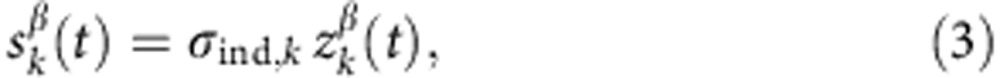


where *c* is the stimulus coherence and *γ*^*β*^ the average additional input at highest coherence *c*=1. Without loss of generality, we assume that the stimulus is moving in the preferred direction of E1 neurons, that is, we use a positive *γ*^E1^ and a negative *γ*^E2^ with *γ*^E2^=−*γ*^E1^, so that the firing rates of E1 (E2) neurons increase (decrease) approximately linearly with *c*, as observed experimentally^36^. Temporal modulations in sensory input generated by the specific realization of the dot trajectories in RDKs are captured by the time-varying terms *z*^*β*^(*t*) and 
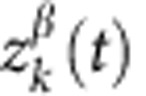
 (independent Ornstein-Uhlenbeck processes with zero mean, s.d. equal one and time constant *τ*_stim_=20 ms). The amplitude of the temporal modulations is set by *σ*_stim_=*σ*_ind,*k*_=0.212 *σ*, where *σ* is the dimensionless strength of stimulus modulations (except when otherwise indicated).

Repeated presentations of the stimulus over trials were done in two ways: (1) in the replicate stimulus condition we injected the exact same realization of stimulus currents 
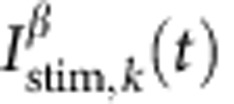
 into each cell in every trial, (2) in the non-replicate stimulus condition we injected different realizations of the currents 
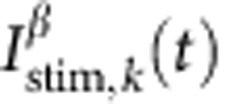
 in every trial, with different realization of both *s*^*β*^(*t*) and 
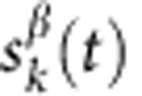
, what caused trial-to-trial fluctuations in the stimulus input. We modelled the stimulus as an injected current instead of a barrage of pre-synaptic spikes so that sensory input *per se* did not constitute an uncontrolled source of variability in the replicate stimulus condition.

### Simulation details

The network model was implemented in Python using the Brian simulator version 1.4 (ref. 63). The network model code is available at ModelDB (https://senselab.med.yale.edu/ModelDB/). We used the Euler integration method with a time step of 0.1 ms. We simulated fixed-duration trials with a stimulus duration of 2 s, as in experimental settings^2^,^9^. Stimulus presentation was preceded by a 3-s interval to prevent transient effects due to initial conditions. The choice outcome of the network was determined by the population of the integration circuit (D1 or D2) with a higher population firing rate over the last 50 ms of the stimulus period. Results for a given parameter set are based on 2,000 repeated trials of the same network (same connectivity matrix) with random initial conditions as well as different realizations of the external background inputs into each circuit.

For the replicate stimulus condition (see above) we generated 100 distinct realizations of replicate stimuli and presented each of them over 100 repeated trials (in total 10,000 trials). Replicate stimuli that led to overly consistent responses (>95 % choices in one direction) were excluded from the analysis because there were too few trials yielding one of the choices to have a good estimate of CP (this was the case for 23 of the 100 replicate stimuli).

Slower decision dynamics (Fig. 3c) was realized by decreasing the efficacy of feedforward connections 
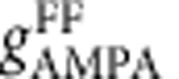
 from the sensory to the integration circuit by 25% and increasing the temporal modulations of the stimulus (*σ*=1.33). This led to a longer ‘sensory integration window’ (1.057 s versus 0.675 s; see below).

The heterogeneous network (Figs 5 and 6 and Supplementary Figs 9-10) is identical to the homogeneous network, but not all sensory neurons receive stimulus and top-down inputs. We randomly split each sensory population in four neural groups of equal size that receive (1) both stimulus and top-down feedback inputs (S+FB+), (2) only stimulus (S+FB−), (3) only top-down (S−FB+) and (4) neither stimulus nor top-down (S−FB−). This was achieved by using an individual feedback strength 
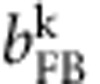
 and an individual amplitude of input modulations *σ*_stim, *k*_ for each neuron *k*. We set 
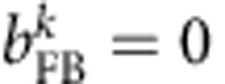
 (no top-down) for neurons in FB− and 
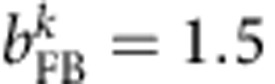
 (strong top-down) for neurons in FB+. We set *σ*_stim,*k*_=0 and 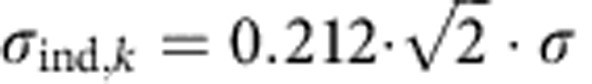 for neurons in S−, and 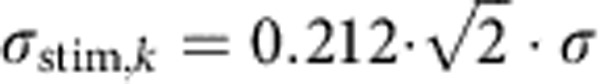 and *σ*_ind,*k*_=0 for neurons in S+. This yields different (identical) input currents into each cell in S− (S+), without changing the s.d. of input currents 
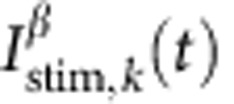
 compared with the homogeneous network (see equation (1)). All other parameters were left unchanged.

### Physiological data

Physiological recordings had previously been obtained by K.H. Britten, J.A. Movshon, W.T. Newsome, M.N. Shadlen and E. Zohary. Experimental details are described in refs 2, 13, 64. In brief, three adult macaque monkeys (*Macaca mulatta*, two male and one female) performed a fixed-duration motion direction discrimination task near psychophysical threshold while responses of single neurons^2^,^64^ or pairs^13^ in MT/V5 were recorded. The stimuli, RDKs at various motion coherences, were matched to each neuron’s preference for stimulus size, speed and motion direction. The precise pattern of random dots of the kinematograms at each coherence was either different (non-replicate RDK) or the same across trials (replicate RDK). The experimental data sets are available in the Neural Signal Archive (www.neuralsignal.org; single units: nsa2004.1 and nsa2009.1; paired units: nsa2004.2 and nsa2012.1). Most single units were recorded either in the non-replicate or replicate condition, but a subset of 22 neurons was recorded under both conditions. For these neurons, the impact of stimulus fluctuations on Fano factor and CP was consistent with the data shown in Fig. 4a,c: the shift-corrected Fano factor was higher in the non-replicate than in the replicate condition, and CP was higher in the non-replicate compared with the replicate condition early (*P*=0.05), but not late (*P*=0.70) for a count window *T*=250 ms. Paired units were only recorded in the non-replicate condition, except for two neural pairs (emu034 and emu035) that were obtained for both replicate and non-replicate stimuli (Fig. 4b and Supplementary Fig. 8b).

We used recordings from two monkeys (E and W) and excluded data from a third monkey (J) because the average CPs (*T*=2 s) obtained in this monkey were only marginally above chance level^2^ and were significantly smaller than for the other two monkeys (one-way ANOVA, F_(2,250)_=6.71, *P*=0.0015; mean CP was 0.565±0.010 for monkey E with *n*=117, 0.561±0.012 for monkey W with *n*=67, and 0.509±0.012 for monkey J with *n*=67). Monkey J also had considerably higher psychophysical and neuronal thresholds than E and W^64^. To be included in the analysis, neurons had to fulfil the following criteria: (1) more than 20 trials are available for the zero-coherence condition (2) in these trials there are at least five preferred and five non-preferred choices, (3) the average firing rate is higher than 1 sp s^−1^, and (4) the neuron fired at least 100 spikes across all trials. Outlier trials in which the spike count deviated from the mean by more than 3 s.d. were excluded. Neurons from the paired-unit data set nsa2004.2 whose preferred direction differed by <35° were included in the single unit analysis (47 neurons from monkey E, all with non-replicate stimuli). This ensured that the direction of the stimulus did not differ from the preferred orientation of the neurons, causing a decrease in the magnitude of CP^10^. For the analysis of pairwise correlations (Fig. 6) we used 32 pairs (all from monkey E) whose preferred directions differed by <90°.

### Spike count statistics and choice probabilities

After binning time using d*t*=1 ms, the spike train of neuron *k* in trial *l* is represented as a binary word 
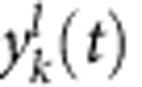
 that equals 1 if there is a spike in the interval *(t*, *t+*d*t)* and zero otherwise. The instantaneous spike count 
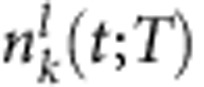
of neuron *k* in trial *l* over a count window (*t*–*T*/2, *t+T*/2) is defined as:


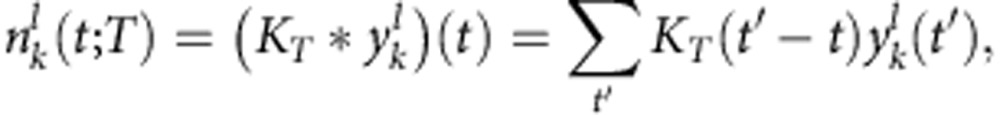


that is the discrete convolution of 
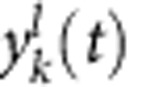
 with the kernel *K*_*T*_(*t*) which equals one in (–*T*/2, *T*/2) and zero otherwise.

The individual trial-averaged rate of neuron *k* (Supplementary Figs 1a-c and 8a) is defined as 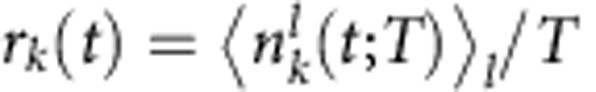 where the brackets 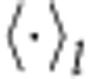 represent the average over trials and *T*=50 ms. The instantaneous activity of population *β* in trial *l* (Figs 1b and 7a,b) is defined as 
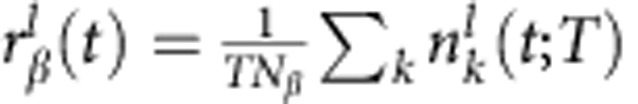
 with the sum running over the *N*_*β*_ cells of population *β* (*T*=50 ms). The population rate averaged over the trials yielding choice *α* (with *α*=1,2) is defined as 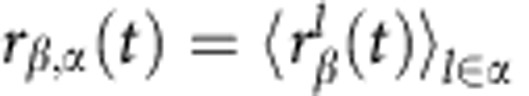. We label *r*_*β*,*α*_(*t*) as preferred and non-preferred for *α*=*β* and *α≠β*, respectively (Fig. 2a,d and Supplementary Fig. 3a).

The instantaneous CP *CP*_*k*_(*t*;*T*) of neuron *k* is obtained by classifying the spike counts across trials 
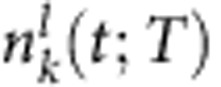
 according to the choice yielded in each trial (that is,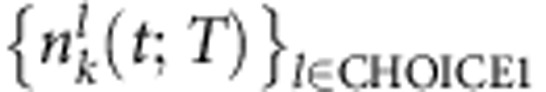 versus 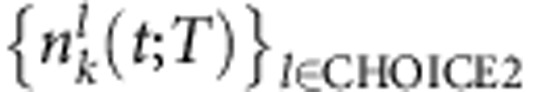). The *CP*_*k*_(*t*; *T*) is defined as the area under the receiver operating characteristic curve obtained from these two distributions^2^.

The spike count Fano factor for neuron *k* (Fig. 4a) is defined as the ratio of the spike count variance to the mean spike count:


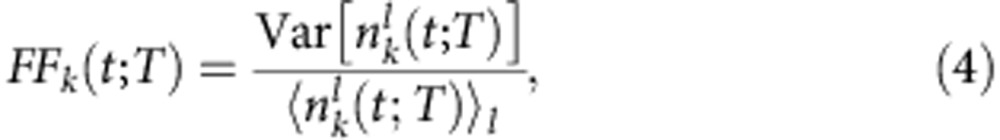


where the variance is obtained over trials.

Spike count noise correlations were measured using the Pearson correlation coefficient of the spike counts of neuron *k* and *k′* at times *t* and *t′*:


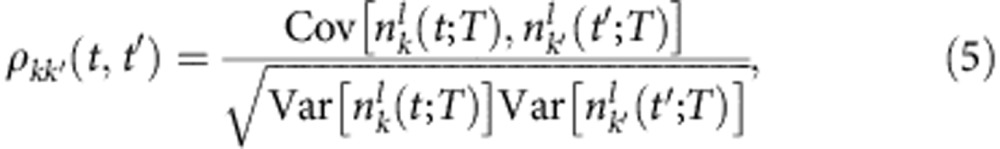


with the covariance and the variance obtained across trials (we have dropped the explicit dependence on *T* to ease the notation). The average over the population of pairs (*k*, *k′*) was denoted as *ρ*(*t*, *t*′) (Fig. 6a–g). We defined the instantaneous (non-lagged) correlation as 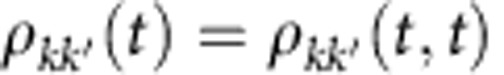. The correlation matrices *ρ* (*t*_*i*_, *t*_*j*_) in Fig. 6a–c were obtained by evaluating *ρ*(*t*, *t*′) at *t*_*i*_=(*i*−1/2) *T* and *t*_*j*_=(*j*−1/2) *T*, with *i*, *j*=1*…*8 and *T*=250 ms. Finally, we represented the diagonals of *ρ*(*t*_*i*_, *t*_*j*_), defined as constant, versus the time lag *t*_*i*_–*t*_*j*_ as a way to visualize an instantaneous cross-correlogram (Fig. 6a,b insets).

To remove a potential influence of differences in the average spike count on the measured Fano factor^65^, correlation and the CP^12^,^66^, we used adjusted count windows of variable length to compute *FF*(*t*), *ρ*_*kk*′_(*t*) and *CP*(*t*) (Fig. 4a–c). The spike count window for each cell *k* centred at each time point *t* was adjusted to (*t*−*T*′/2, *t*+*T*′/2) to contain exactly *n*_*k*_ spikes on average (across trials). The number *n*_*k*_=*r*_*k*_*T*, where *r*_*k*_ is the trial-averaged rate over the stimulus duration (2 s). The Fano factors *FF*(*t*) and correlations *ρ*_*kk*′_(*t*) were very similar for fixed and adjusted count windows, as was the *CP*(*t*) in the non-replicate condition. For the replicate condition, where the trial-averaged rate shows strong temporal modulation^28^ (see Supplementary Fig. 8a), CP(*t*) was smoother for adjusted windows. The finding that early *CP*(*t*) decreases in the replicate condition does not depend on the count window *T* (Fig. 4c) or whether we used fixed or adjusted count windows.

For the network model, we averaged *CP*_*k*_(*t*; *T*) and *ρ*_*kk*′_(*t*_*i*_, *t*_*j*_) over 100 randomly chosen neurons from populations E1 and E2 (or over all the pairs formed by these neurons) with a minimum firing rate of 1 sp s^−1^. For the experimental data we averaged *CP*_*k*_(*t*; *T*), *FF*_*k*_(*t*; *T*) and *ρ*_*kk*′_(*t*, *t*′) over a variable number of neurons and pairs (see legends of Figs 4, 5, 6 and Supplementary Fig. 8). Data analysis was restricted to trials with zero-coherence stimuli, except for the correlation measurements. Correlations *ρ*_*kk*′_(*t*) of single pairs (Fig. 4b and Supplementary Fig. 8b) were calculated separately for the available stimulus coherences ranging from –51.2% to +51.2% and then averaged (negative coherences represent motion in the non-preferred direction). Correlation matrices *ρ*(*t*_*i*_, *t*_*j*_) (Fig. 6) were obtained using low motion coherences (–3.2, 0 and +3.2%) that yielded a comparable number of trials for each choice. This gave a total of n=64 conditions from the 32 cell pairs.

When analysing the experimental data, we modified equations (4) and (5) to obtain Fano factors and correlations to remove the impact of slow variations in firing rate across trials^25^. We used the shift-corrected spike count covariance and variance defined as:


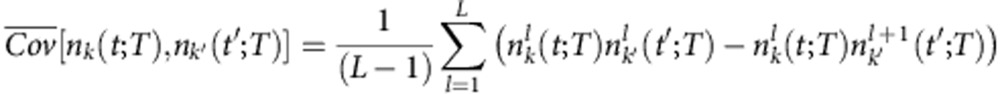


and


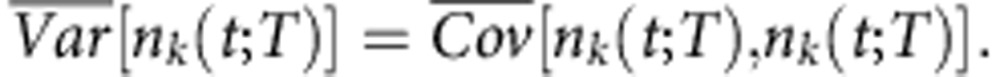


Results for Fano factors are shown using this correction. For spike count correlations we did not use the correction because it did not affect the estimation at small spike count window *T* (<250 ms) but yielded larger estimation errors for large *T*. Results for Fano factors and correlations did not qualitatively change with the application of the shift correction.

In the analysis of the dependence of CPs, Fano factors and correlations on *T* (Fig. 4, Supplementary Figs 4 and 8), we averaged *CP*_*k*_(*t*; *T*), *FF*_*k*_(*t*; *T*) and *ρ*_*kk*′_(*t*) across the time points *t=T/2*, *3/2 T*, *5/2 T*, *…* so that the statistics come from non-overlapping spike count windows starting at stimulus onset (*t*=0). We did this for *T=*15, 30, 60, 125, 250, 500, 1,000 and 2,000 ms.

The CP correlation matrix (Fig. 5 and Supplementary Fig. 9) is defined as the Spearman’s rank correlation coefficient across neurons of the CP measured at two different time bins:


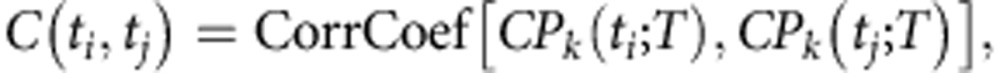


with *CP*_*k*_(*t*; *T*) evaluated at times *t*_*i*_=(*i*−1/2) *T* and *t*_*j*_=(*j*−1/2) *T*, with *i*, *j*=1*…*8 and *T*=250 ms. The matrix *C(t*_*i*_, *t*_*j*_) was obtained for the network (Fig. 5b,d and Supplementary Fig. 9) using a similar number of neurons and trials as available in the MT data. We selected 160 neurons (40 of each group) and computed their CP time-courses based on *n* randomly selected trials (with *n*=100 in Fig. 5b,d; *n*=100, 200 and 2,000 trials in Supplementary Fig. 9). We calculated *C(t*_*i*_, *t*_*j*_) as the average across 1,000 different selection of trials from which s.e. values were obtained. All data analyses were carried out in MATLAB (The Mathworks).

### Psychophysical data

Two adult macaque monkeys (*Macaca mulatta*, male) were trained to report with a reaching response the motion direction of a random dot kinematogram (RDK, see Supplementary Methods) along the horizontal axis with varying levels of motion coherence. On each trial we recorded both the monkey’s choice and the presented stimulus (that is, the dots positions in each frame). These data were used to compute average motion energy traces (Supplementary Fig. 7). The task was very similar to the classical fixed-duration version^2^,^13^,^64^ (see Supplementary Methods for details). All surgical and behavioural procedures conformed to guidelines established by the National Institutes of Health and were approved by the Institutional Animal Care and Use Committee of Stanford University.

### Psychophysical reverse correlation

We used psychophysical reverse correlation^9^,^67^ to measure the amplitude and time-course of the impact of stimulus fluctuations on the decision. The psychophysical kernels were computed as the difference of the average stimulus leading to each of the two possible choices. For the experimental data, stimulus fluctuations were estimated by computing the motion energy contained in the RDKs using appropriate spatio-temporal filters^29^,^68^. For details, see Supplementary Methods.

## Author contributions

J.R., A.C. and K.W. conceived the study and developed the computational model. All the authors contributed to the design of the study and to the interpretation of the data. D.P. collected the psychophysical data; K.W. performed the simulations and analysed the simulation and psychophysical data; K.W. and J.R. analysed the physiological data; K.W. and J.R. wrote the paper, with contributions from the rest of the authors.

## Additional information

**How to cite this article:** Wimmer, K. *et al*. Sensory integration dynamics in a hierarchical network explains choice probabilities in cortical area MT. *Nat. Commun.* 6:6177 doi: 10.1038/ncomms7177 (2015).

## Supplementary Material

Supplementary Information Supplementary Figures 1-10, Supplementary Tables 1-4, Supplementary Methods and Supplementary References
