# Supplementary material for: Sensory integration dynamics in a hierarchical network explains choice probabilities in cortical area MT
Source: Nat Commun. 2015 Feb 4;6:6177. doi: 10.1038/ncomms7177 (PMC4347303; doi:10.1038/ncomms7177)
Supplement: Supplementary Information — Supplementary Figures 1-10, Supplementary Tables 1-4, Supplementary Methods and Supplementary References [file ncomms7177-s1.pdf]

Dynamics of sensory integration in a hierarchical network explains choice probabilities in cortical area MT

SUPPLEMENTARY INFORMATION

Klaus Wimmer, Albert Compte, Alex Roxin, Diogo Peixoto, Alfonso Renart & Jaime de la Rocha

Supplementary Figures

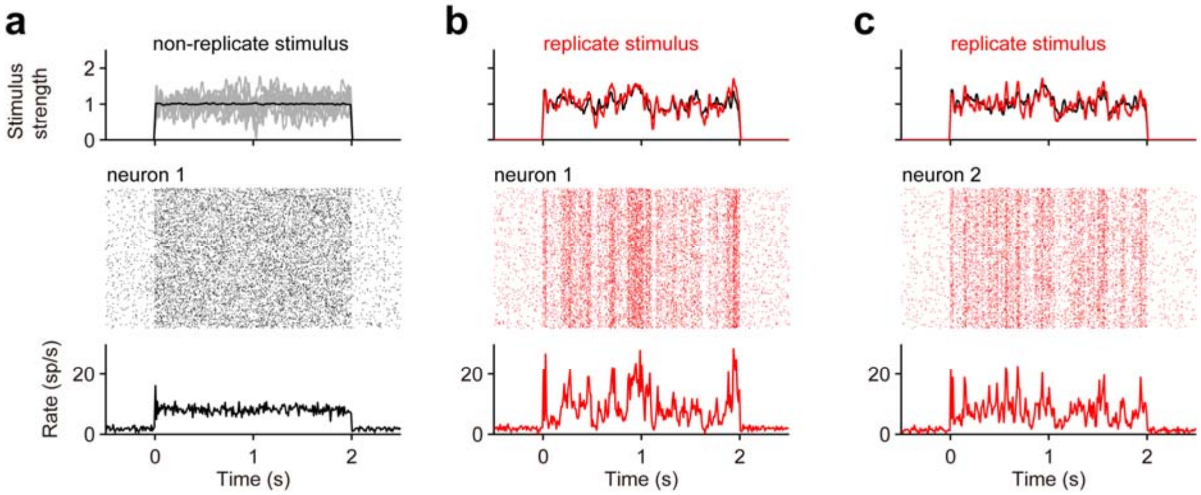

Supplementary Figure 1. Replicate vs. non-replicate conditions in the network model. (**a**) Stimulus and responses of an example neuron in the non-replicate condition (different stimuli across trials). Top: stimulus injected current from several individual trials (gray) and averaged across trials (black). The rastergram from 500 trials and the corresponding PSTH (T = 20 ms) are shown below. (**b-c**) Stimulus and response of two example neurons from the same population in the replicate condition (identical stimuli across trials). Top: common term of the stimulus $I_c^{\beta}(t)$ (black; same for both neurons in **b-c**) and the individual stimulus realization $I_{\text{stim},\,k}^{\beta}(t)$ (red) for neurons k = 1,2 (see Methods). Individual stimulus currents injected into cells within the same population had a temporal correlation coefficient $\text{CorrCoef}\left[I_{\text{stim},k}^{\alpha}(t), I_{\text{stim},k'}^{\alpha}(t)\right] = 0.5$ on average. Bottom: rastergrams from 500 trials in response to the same stimuli $I_{\text{stim},k}^{\beta}(t)$ and the corresponding PSTHs.

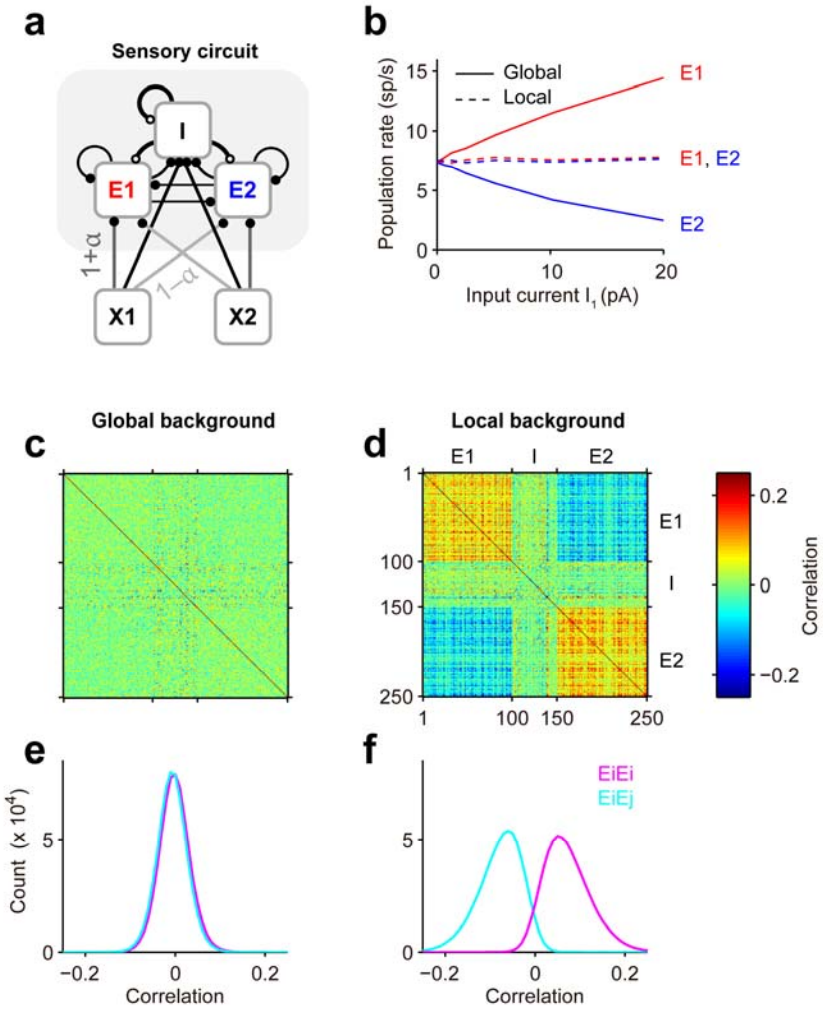

Supplementary Figure 2. Structure of bottom-up correlations in the sensory circuit. (**a**) Parametric variation of the spatial arrangement of the external background inputs using two separate external populations X1 and X2 (rather than one common population X). The connection probability from population Xi to Ej is $p_{XiEj} = p_x(1+\alpha)$ for $i = j$ and $p_{XiEj} = p_x(1-\alpha)$ for $i \neq j$. The parameter α varies between 0 (global background input, standard network as in **Fig. 1a**) and 1 (local background input, where each Ei receives specific connections from its corresponding external population Xi). The connection probability from X1 and X2 to population I is p_x . (**b**) Response rates of the sensory populations to a global (dashed lines) and a local (solid lines) external input. We injected a constant input current I_1 into both E1 and E2 (global), or an input $2I_1$ into E1 only (local). The inhibitory population received I_1 in both cases. Excitatory neurons also received a baseline input of I_0 = 80 pA, equivalent to the mean input of zero-coherence stimuli (Methods). A global increase in the input leads to a marginal increase of the rates r_{E1} and r_{E2} . In contrast, a local increase of the input to E1 yields a substantial increase of r_{E1}, and, due to the strong common inhibition, a corresponding decrease in r_{E2}. This is the mechanism underlying competitive dynamics in the circuit. (**c-f**) Spike count correlation matrices $\rho_{kk'}$ (**c-d**) and correlation histograms (**e-f**) of the sensory circuit obtained for global ($\alpha = 0$) or partly local ($\alpha = 0.67$) background inputs. Color plots show the correlation matrices from 100 + 100 + 50 randomly picked neurons from the E1, E2 and I populations, respectively. Histograms are shown for within (EiEi) and across population (EiEj) pairs. Correlations were obtained from the response to replicate stimuli (count window T = 2 s). Correlations in a pair EiEj caused by a global ($\alpha = 0$) or local ($\alpha > 0$) fluctuating input are proportional to

the product of the slopes $\frac{dr_{Ei}}{dI_1} \cdot \frac{dr_{Ej}}{dI_1}$ obtained using global or local constant input I_1 (**b**; ref. [1]). This explains why local background inputs induce positive EiEi and negative EiEj correlations with average correlation across all EE-pairs close to zero[2]. Correlations EI and II also had a mean close to zero (not shown). This mechanism also explains why non-replicate stimuli or top-down inputs, which can be both viewed as local inputs into E1 and E2 that fluctuate from trial-to-trial, generate correlations with the same spatial structure as local background inputs (compare **Fig. 2c,f** with **Supplementary Fig. 3c**).

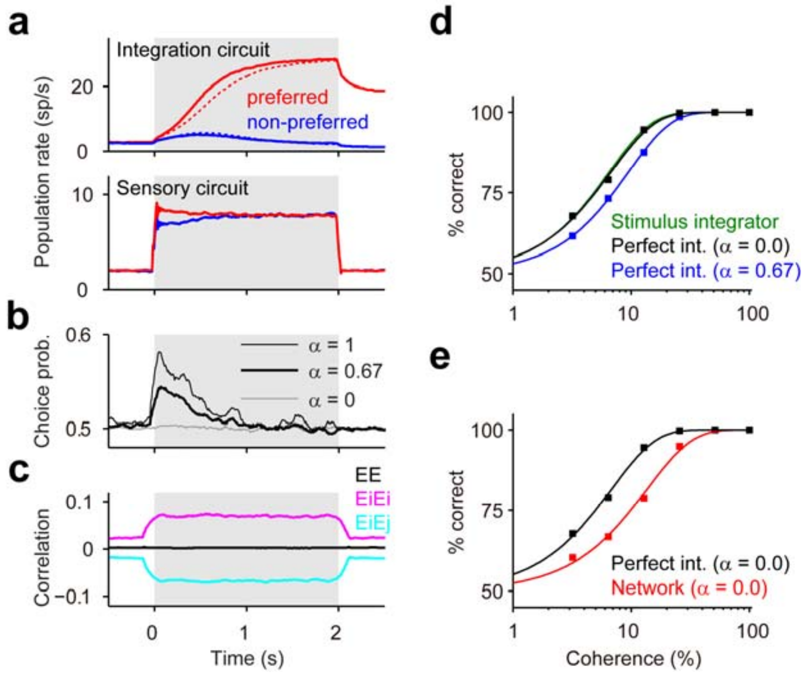

Supplementary Figure 3. Impact of bottom-up correlations and sensory integration dynamics on choice probability and categorization accuracy. (**a-c**) Average population rates for preferred and non-preferred choice trials in the integration and sensory circuits (**a**), average CP (**b**) and spike count correlations (**c**) obtained in a network with spatially localized external background input ($\alpha = 0.67$, except in **b**) using replicate stimuli and top-down connections set to zero ($b_{FB} = 0$). Correlations were sustained and had a structure among sensory neurons similar to that caused by trial-to-trial stimulus fluctuations (compare **c** to **Fig. 2c**). In consequence, they produced a CP with the fast-rise-decay time-course described in **Fig. 2b**. Shaded areas in **a-c** represent the stimulus interval. (**d**) Percentage of correct categorizations versus stimulus coherence when stimuli are classified according to their accumulated evidence for motion in the β-direction (by computing the integrals $\int s^{\beta}(t)$ of stimulus inputs into populations β = E1, E2; "Stimulus integrator") or by using a perfect integrator reading out the sensory activity (see **Supplementary Figure 5**). For α = 0, the perfect integrator reached the accuracy of the stimulus integrator. Noise correlations introduced by $\alpha > 0$ had a structure similar to signal correlations (**Supplementary Fig. 2d**) and for that reason they decrease discrimination accuracy[3,4]. Note that due to temporal stimulus modulations (σ > 0), a fraction of low-coherence stimuli actually provides accumulated evidence favoring the opposite direction and this limits the accuracy that can be reached. Although trial-to-trial fluctuations caused by non-replicate stimuli can generate a similar structure of correlations than local background inputs, they do not cause a decrease in discrimination accuracy because they are not truly *noise* correlations but spurious *signal* correlations. (**e**) Percentage of correct categorizations as a function of stimulus coherence for the perfect integrator (black; replotted from **d**) and for the hierarchical network model (red) when $\alpha = 0$. Because the network performs a transient integration of the sensory information (i.e. non-uniform across time, **Fig. 3b**) it yields a lower classification accuracy than the perfect integrator (see also **Figure 7h**). Simulation data (squares) were fitted using a Weibull function (solid lines).

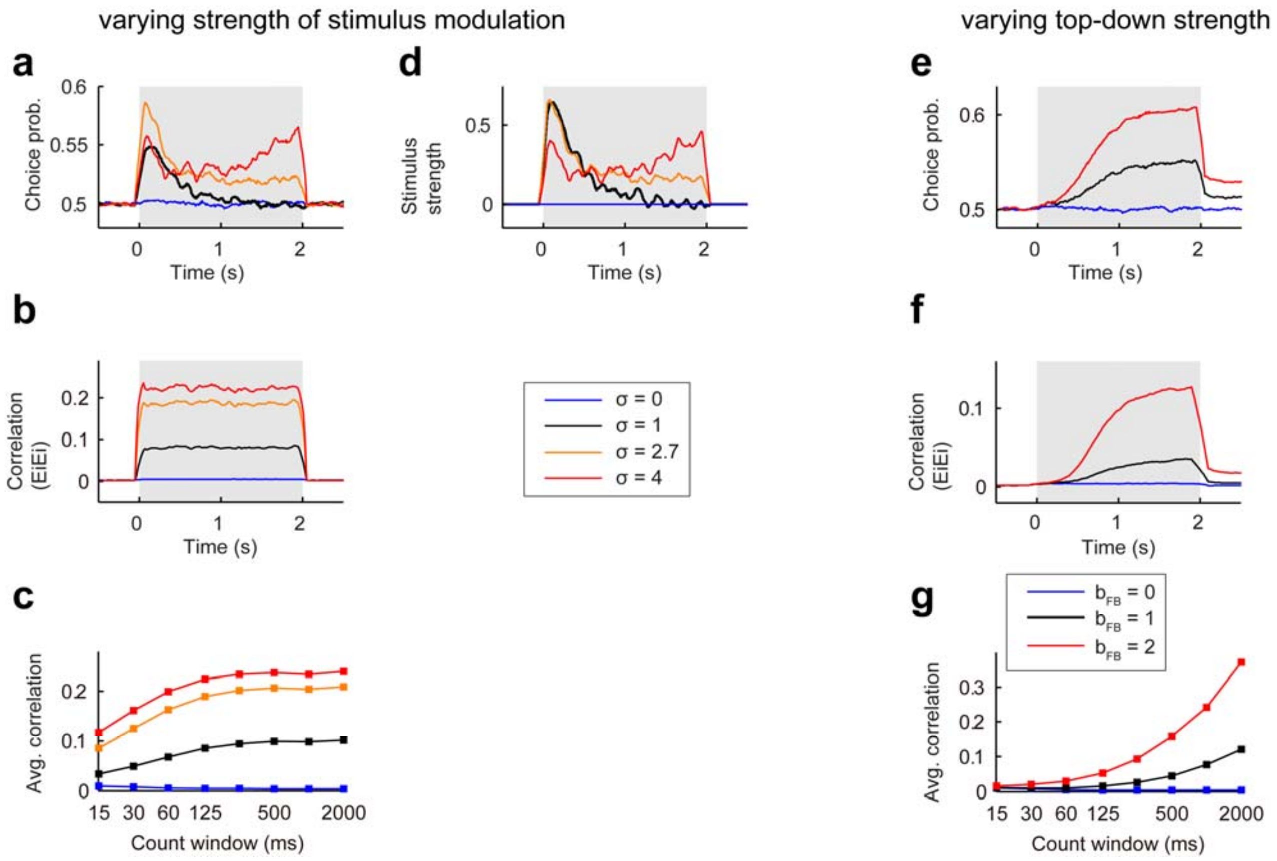

Supplementary Figure 4. Impact of stimulus modulation amplitude and strength of top-down signals on choice probability and correlations in the network model. (**a**) Average CP of sensory neurons obtained using non-replicate stimuli at various modulation amplitudes σ in the network without top-down connections (count window T = 100 ms). CP for $\sigma = 1$ is also shown in **Fig. 2b**. (**b-c**) Average spike count correlations of pairs of excitatory sensory neurons within the same population (EiEi) versus time (**b**; T = 100 ms) and the dependence of the time-averaged correlation on the count window size T (**c**). The plateau obtained for T > 125 ms indicates that correlations produced by the stimulus fluctuations exhibited a fast time-scale. (**d**) Psychophysical kernels (difference of average choice-conditioned stimuli) for various σ. Trial-to-trial stimulus fluctuations of moderate amplitude ($\sigma \sim 1$) generate positive average EiEi correlations (**b-c**) and a fast-rise-and-decay CP (**a**). For large stimulus modulation ($\sigma = 2.7$ and 4), the CP and the psychophysical kernel do not decay to baseline, indicating that stimulus modulations are strong enough to revert the categorization even after the integration circuit has reached a choice attractor (see **Fig. 7**). For larger σ values (> 4) the winner-take-all dynamics in the integration circuit are overridden by the large sensory modulations. The integration circuit does no longer accumulate evidence over the stimulus but works as a memoryless leaky integrator yielding a CP and a psychophysical kernel with a ramping-up time-course[5] (not shown). Note the similarity in the time-course of CP and the psychophysical kernel across σ values characteristic of a network without top-down connections. (**e**) Average CP obtained for various top-down connection strengths b_{FB} (see values in **g**) using replicate stimuli ($\sigma = 1$ as in **Fig. 1b**; T = 100 ms). (**f-g**) Average correlations of pairs within one population (EiEi) versus time (**f**, T = 200 ms) and the dependence of the time-averaged correlation on T (**g**). The increase of the correlation with T over the entire range 15 - 2000 ms reflects that the trial-to-trial correlations induced by the top-down feedback have a slow time-scale. Shaded areas represent the stimulus interval.

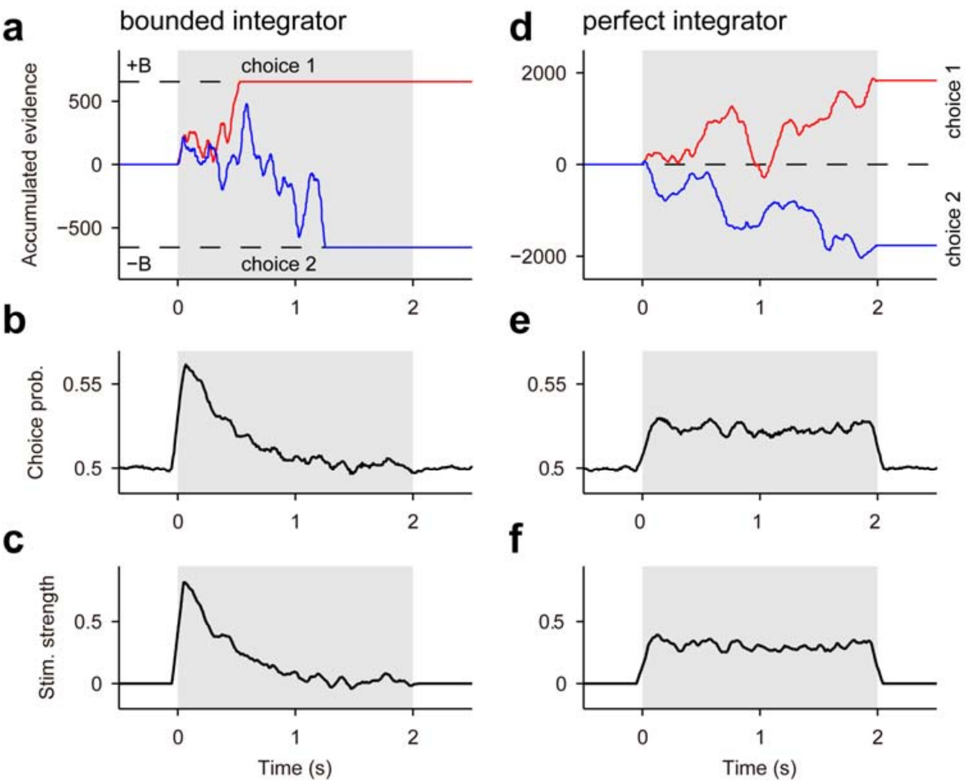

Supplementary Figure 5. Choice probability and psychophysical kernel for bounded integrator and perfect integrator models. (**a**) The *bounded integrator model* integrates evidence by continuously "summing up" the spikes fired by each sensory population, with weights +1 and –1 for each spike from neurons in populations E1 and E2, respectively. The accumulation process terminated when the integration variable reached the absorbing bounds at +B or −B (dashed lines), after which it remained constant and impervious to sensory activity. Traces from two example trials yielding choice 1 (red) and choice 2 (blue) are shown. (**b-c**) Both CP and psychophysical kernel obtained using the bounded integrator show a fast-rise-and-decay time-course, quantitatively similar to those obtained in the network with the accumulation dynamics implemented by the spiking attractor model (**Fig. 2b** and **Fig. 3b**, respectively). (**d-f**) As in **a-c** but for the *perfect integrator model* where evidence is accumulated during the whole stimulus period (no bounds). The decision is determined by the sensory population which fired more spikes during the entire period. Traces show the integration variable in two example trials yielding choices 1 (red) and 2 (blue). The dashed line indicates the decision boundary. Both the CP (**e**) and the psychophysical kernel (**f**) show a sustained time-course. This indicates that the impact of sensory evidence on the decision is constant across time, which is at odds with the experimentally observed decay in the psychophysical kernel derived from RDKs (**Supplementary Fig. 7c,e**). For both integrator models we employed the spikes generated by the sensory circuit in the simulations used to generate **Fig. 2a-c**, i.e. the network without top-down connections receiving non-replicate stimulus input. Shaded areas represent the stimulus interval.

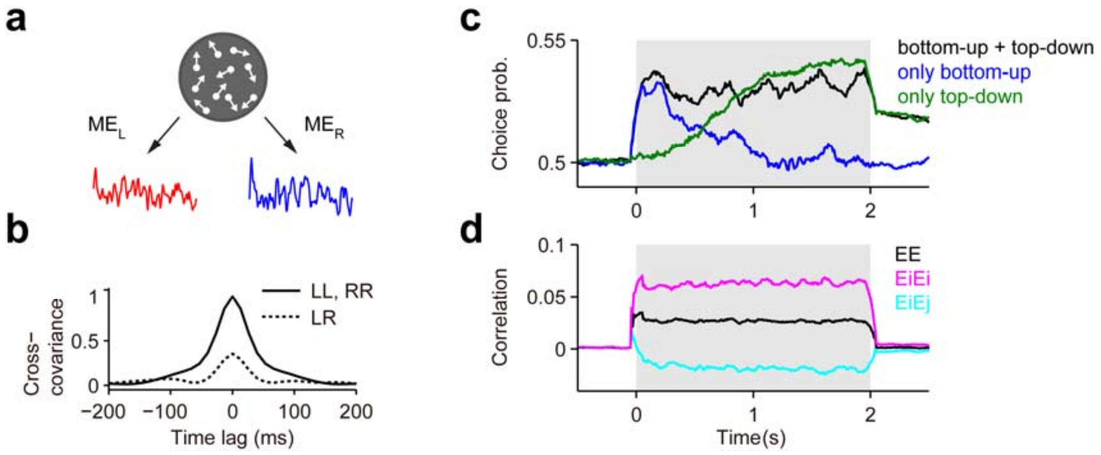

Supplementary Figure 6. Bottom-up correlations together with top-down signals can lead to sustained CP in the presence of increased average correlations between sensory neurons. (**a**) Illustration of the time-course of oriented motion energy in opposite directions calculated from a zero-coherence RDK (ME_L and ME_R are the left and right motion energy, respectively). Motion energy traces are obtained by: (1) filtering the RDK with a quadrature pair of spatio-temporal filters, (2) calculating local motion energy by summing the squared filter outputs, and (3) averaging local motion energies across space[5,6] (see Methods). (**b**) Auto-correlation function of motion energy traces (LL, RR, solid line) and cross-correlation between motion energy traces in opposite directions (LR, dotted line). The cross-correlation at time lag 0 corresponds to the Pearson correlation coefficient r_{LR} between the left and right motion energy traces ($r_{LR} = 0.35$). This positive correlation is a consequence of weak responses of the spatio-temporal filters in the reverse direction[6]. Overall, the fluctuations in motion energy, obtained using filters with a passband matched to the speed of coherent motion (Methods), are correlated on a relatively short time-scale of ~50 ms. (**c**) We mimic the positive correlation between opposite motion energies by introducing a positive correlation in the stimuli injected to populations E1 and E2. This led to an increase in the average spike count correlations across all pairs (black trace in **d**) but similar CP as in the network with independent left and right stimulus inputs. The network is identical to the one used for **Fig. 3a**, except that: (1) Competition between sensory populations E1 and E2 was decreased by removing the potentiation (suppression) of synaptic connections between (across) populations by setting $w_+ = w_- = 1.0$ (Methods). (2) The stimulus currents $I_c^{\beta}(t)$ into neurons of populations $\beta = (\mathrm{E1}, \mathrm{E2})$, that represent oriented motion energy, were correlated ($r = 0.5$) rather than independent. Average CP caused by non-replicate stimuli and top-down inputs ($\sigma = 2.7$, $b_{FB} = 2$; black) is sustained during the stimulus interval. Two complementary contributions to CP are revealed by removing trial-to-trial stimulus fluctuations (i.e. using replicate stimuli; green) or by removing the top-down connections ($b_{FB} = 0$; blue). Thus, the results of **Fig. 3**, obtained with a network with almost zero average correlations across all pairs, are qualitatively unchanged if overall average correlations are larger but the correlation difference corr(EiEi) – corr(EiEj) is maintained. (**d**) Average spike count correlations of sensory neurons (T = 100 ms). Due to the correlated input, correlations are positive in pairs of neurons within the same population (EiEi, pink), slightly negative across populations (EiEj, cyan), and positive across all pairs (EE, black).

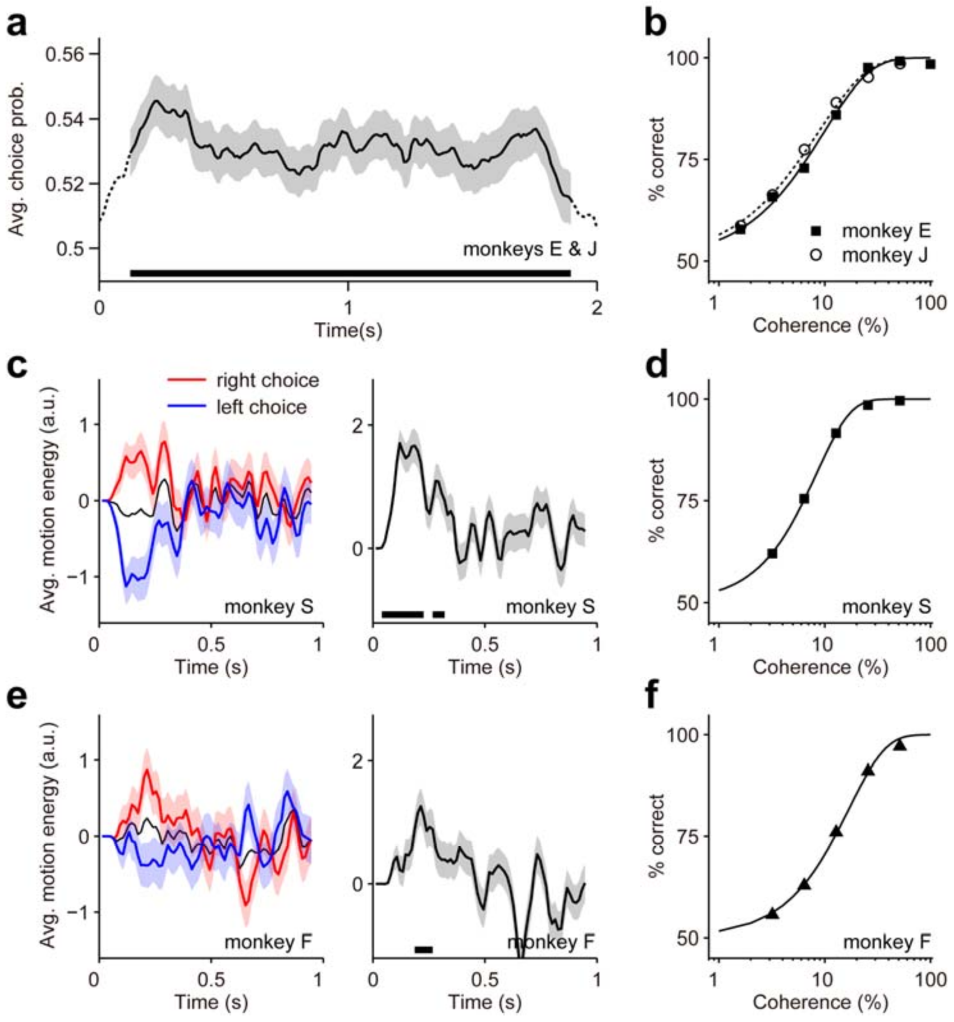

Supplementary Figure 7. Different time-course of MT choice probability and the psychophysical kernel in the random dot direction discrimination task. (**a**) Average CP obtained during a fixed 2-second duration motion discrimination task (n = 143 neurons from monkeys E and W[7,8], count window T = 250 ms). Count windows partially off the stimulus interval are indicated by dotted lines. (**b**) Psychometric functions for the two monkeys. Data points were fitted by Weibull functions. Coherence threshold α, defined by 82% correct, was 10.8% and 8.6%, and slope β was 0.98 and 0.93 for monkeys E and W, respectively. (**c**) Left: Average motion energy traces for right choice (red, $n = 861$), left choice ($n = 724$) and all trials (black) from monkey S performing a similar 1-second fixed duration task (Methods). Positive (negative) values indicate rightward (leftward) motion. The stimuli had zero coherence. Right: Difference in motion energy for right vs. left choices. Thick horizontal lines mark periods of significant difference ($P < 0.05$, permutation test). Error bars (shaded regions) indicate s.e.m. (**d**) Psychometric function for monkey S, with threshold α = 8.3% and slope β = 1.34. (**e**) As **c** but for monkey F (622 and 639 left and right choice trials, respectively). (**f**) As **d** but for monkey F (threshold α = 16.7% and slope β = 1.23). Note that all four monkeys show comparable psychophysical performance (monkey F being slightly worse). Thus, transient evidence integration reflected in a decaying psychophysical kernel (**c** and **e**; monkeys S and F) is compatible with high perceptual performance observed for monkeys E and W (**b**) for which the sustained CP was obtained (**a**).

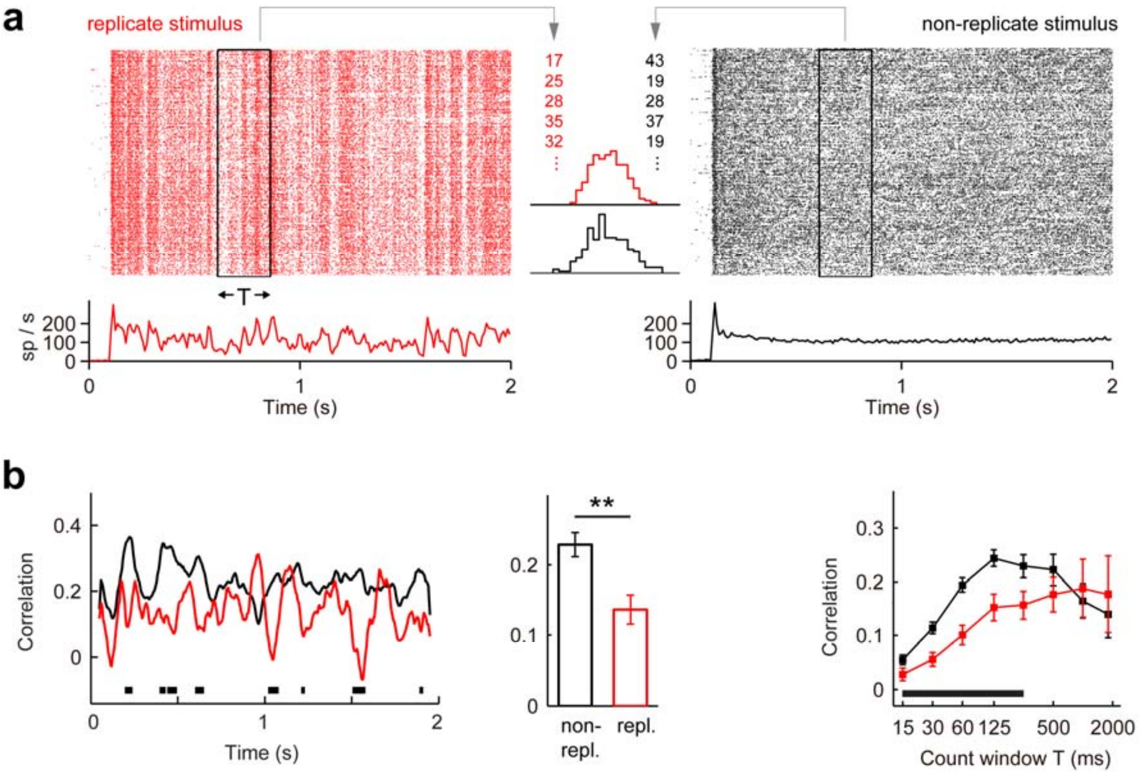

Supplementary Figure 8. Additional data from area MT showing the impact of trial-to-trial stimulus fluctuations on neuronal variability. (**a**) Raster and post-stimulus time histogram (PSTH) for a sample MT neuron (e093) responding to 210 presentations of replicate (red) and non-replicate (black) zero-coherence RDKs (replotted from ref. [9]). The rapid and strong rate surges in the replicate PSTH are presumably induced by random conjunctions of dot trajectories in the preferred direction of this neuron[9,10]. In contrast, the non-replicate PSTH shows no temporal fluctuations because they were averaged out across the ensemble of RDKs. Inset: example spike count histograms from the indicated count windows, with similar mean (replicate: 26.9, non-replicate: 26.7 sp/s) and higher variance in the non-replicate condition (replicate: 32.6, non-replicate: 51.5 sp^2/s^2). The larger variability across trials in the non-replicate condition is due to the trial-to-trial stimulus fluctuations acting as an additional source of variability. (**b**) Spike count correlations for a second pair of neurons (emu034)[8]. Same layout as **Fig. 4b**. Count window T = 100 ms (left and center). Spike count correlations over the whole stimulus period are significantly larger in the non-replicate than in the replicate condition ($P < 0.05$, permutation test), as for the neuron pair in **Fig. 4b**. Note that only 2 pairs have been recorded in both stimulus conditions during the motion discrimination task. Error bars indicate s.e.m. and thick black lines periods of significant differences between the non-replicate and the replicate condition ($P < 0.05$, permutation test).

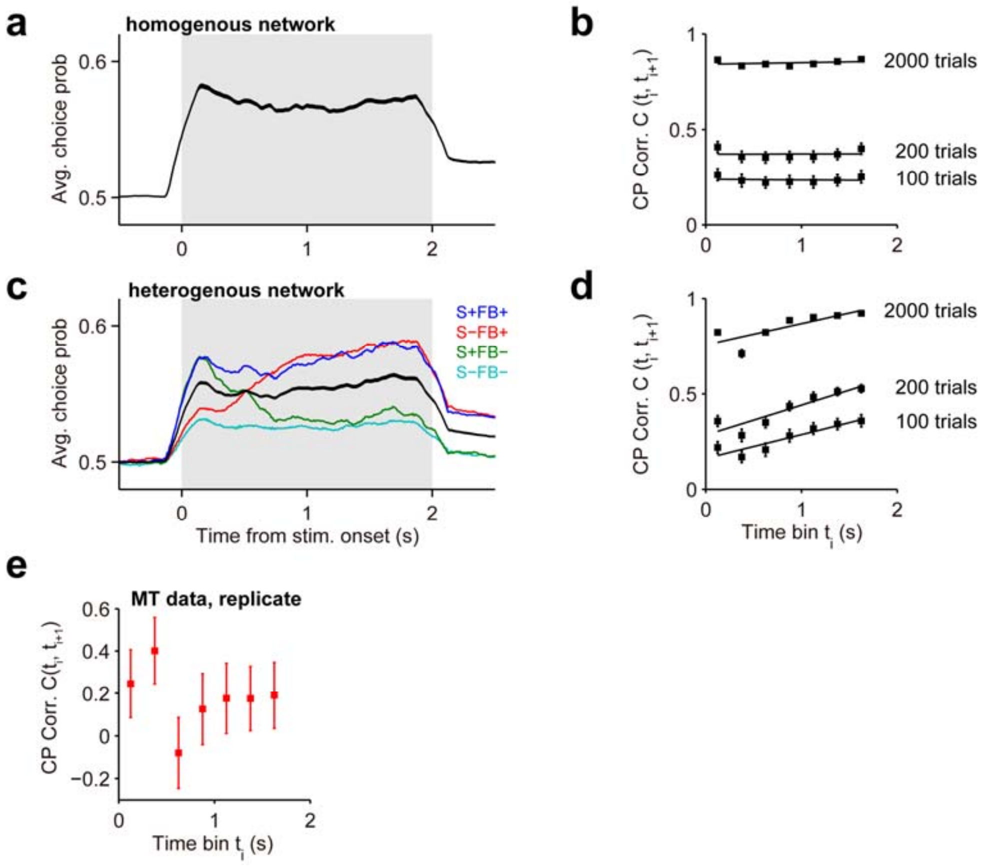

Supplementary Figure 9. CP in the homogeneous and heterogeneous networks, dependence of CP correlations on trial number, and CP correlations in MT for replicate stimuli. (**a**) Population-averaged CP in the homogeneous network (the same network used for **Fig. 5a-b**, count window T = 250 ms) shows a sustained time-course (black line in **Fig. 3a** shows the same model data obtained using T = 100 ms). (**b**) CP correlation $C(t_i, t_{i+1})$ for adjacent time bins (as in **Fig. 5b** right) computed from different number of trials for comparison with data. Increasing the number of trials yields higher $C(t_i, t_{i+1})$ values but does not change the sustained time-course. This shows that the steady behavior of $C(t_i, t_{i+1})$ can be assessed using a small number of trials, comparable to experiments (in the order of 100 trials). (**c**) Average CP (black) across all neurons in the heterogeneous network (the same network as used for **Fig. 5c-d**), and across neurons in each of the four groups (see Methods) that receive (1) both stimulus and top-down inputs (S+FB+), (2) only top-down inputs (S−FB+), (3) only stimulus input (S+FB−), and (4) neither stimulus nor top-down inputs (S−FB−). The average CP of each group is characteristically different, reflecting the hardwired heterogeneity. (**d**) Same as **b**, but for the heterogeneous network. Again, increasing the number of trials yields higher $C(t_i, t_{i+1})$ values but does not perturb the rising of $C(t_i, t_{i+1})$ over time. Thus, the rising of CP correlations is robust to small trial number. (**e**) Time-course of adjacent CP correlations $C(t_i,t_{i+1})$ for the MT data[7] recorded in the replicate condition. Note that statistical power is greatly decreased compared to the non-replicate case (**Fig. 5f**), because here there are only n = 41 neurons and RDKs compared to n = 143 neurons and 25-221 RDKs (median: 59) for the non-replicate case. The CP correlation in the replicate condition shows elevated values early followed by a drop and ramp-and-plateau behavior similar to the non-replicate condition (**Fig. 5f**). The elevated values of CP correlation at the beginning imply that the ordering of early CPs remains fairly stable, although early CPs have a small average magnitude (**Fig. 4c**). A possible explanation is that some neurons exhibit a pre-stimulus CP component, which, due to the absence of a stimulus-driven early CP component, is fairly stable until the top-down component kicks in. In order to investigate why CP correlation is elevated early, we would need more neurons in the replicate condition and pre-stimulus data, which are unavailable in our data set.

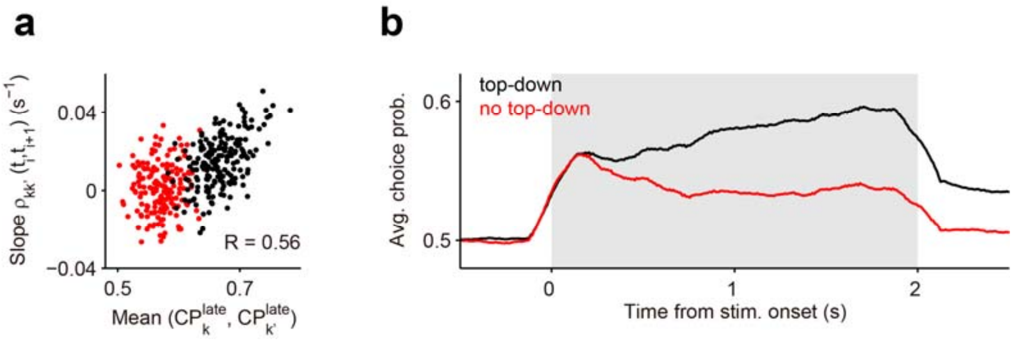

Supplementary Figure 10. Relationship between the dynamics of lagged correlations and late CP in the heterogeneous network model. (**a**) Slopes of the regression of lagged spike count correlations $\rho_{kk'}(t_i,\ t_{i+1})$ against time t_i for individual neuron pairs (T = 250 ms) vs. the mean late CP of the two corresponding neurons calculated over the last second of the stimulus interval (T = 1,000 ms; correlation $R = 0.56$, $P < 10^{-6}$, $n = 357$). Pairs in which both neurons receive top-down connections (black dots; FB+ in **Supplementary Fig. 9c**) tend to have higher slopes of lagged correlations and higher late CP than pairs of neurons that do not receive top-down connections (red dots; FB– in **Supplementary Figure 9c**). (**b**) Average CP time-course for cells that receive (black) or do not receive (red) top-down connections (T = 250 ms). Shaded area represents the stimulus interval.

Supplementary Table 1. Tabular description of the sensory circuit model.

**A: Model Summary**	
Populations	Two excitatory (E1 and E2), one inhibitory (I), one external (X)
Connectivity	Random connections (Erdös-Rényi directed random graph)
Neuron model	Leaky integrate-and-fire, fixed voltage threshold, fixed absolute refractory time; independent fixed-rate Poisson spike trains (external population)
Synapse model	Conductance-based inputs (difference of exponentials PSCs)
Inputs	Stimulus modeled as a time-varying current into excitatory neurons; Top-down feedback signal from neurons in the decision circuit to excitatory neurons
Measurements	Spiking activity

**B: Populations**		
**Name**	**Elements**	**Size**
E1	Iaf neuron	$N_{E1} = 2\ N_I$
E2	Iaf neuron	$N_{E2} = 2\ N_I$
I	Iaf neuron	N_I
X	Poisson	$N_X = 2.5\ N_I$

**C: Connectivity**			
**Name**	**Source**	**Target**	**Pattern**
Local recurrent connections			
EiEj	Ei	Ej	Random with probability p, mean weight $\bar{g}_{EiEj}$, delay d_{E}
EiI	Ei	I	Random with probability p, mean weight $\bar{g}_{EI}$, delay d_{E}
IEj	I	Ej	Random with probability p, mean weight $\bar{g}_{IE}$, delay d_{I}
II	I	I	Random with probability p, mean weight $\bar{g}_{II}$, delay d_{I}
External connections			
XEj	X	Ej	Random with probability p_x, mean weight $\bar{g}_{XE}$, delay d_{XE}
XI	X	I	Random with probability p_x, mean weight $\bar{g}_{XI}$, delay d_{XE}

Connection weights of local recurrent and external connections are drawn from Normal distributions with mean $\bar{g}_{\alpha\beta}$ and standard deviation $\sigma_{\alpha\beta}$. Weights less than zero are set to zero.

Feedback connections from the decision circuit			
FB	Di (decision)	Ei (sensory)	Random with probability p_{FB}, fixed weight g_{FB}, delay d_{FB}

**D: Neuron models**	
Name	Iaf neuron
Type	Leaky integrate-and-fire, conductance-based synaptic inputs
Subthreshold dynamics	$C_m \frac{dV_j^\beta}{dt} = -g_L\left(V_j^\beta - V_L\right) + I_j^{E\beta}(t) + I_j^{I\beta}(t) + I_j^{X\beta}(t) + I_{\mathrm{FB},j}^\beta(t) + I_{\mathrm{stim},j}^\beta(t)$, for $t > t^* + \tau_{\mathrm{ref}}^\beta$ $V_j^\beta(t) = V_R$, otherwise
Synaptic currents	$I_j^{\alpha\beta}(t) = -\sum_i s_{ij}^{\alpha\beta}\left(V_j^\beta - V_{\mathrm{rev}}^\alpha\right)$, where i runs over all presynaptic neurons from population α which have a connection to the postsynaptic neuron j in population β
Spiking	if $V_j^\beta(t^-) < V_T$ and $V_j^\beta(t^+) \geq V_T$: (1) set $t^* = t$ and (2) emit spike with time stamp t^*
Name	Poisson
Type	independent Poisson spike trains with fixed rate v_{ext}

**E: Synapse model**	
Type	Difference-of-exponentials synapse
Synaptic dynamics	$\tau_{\mathrm{decay}} \frac{ds_{ij}^{\alpha\beta}}{dt} = -s_{ij}^{\alpha\beta} + x_{ij}^{\alpha\beta}$ $\tau_{\mathrm{rise}} \frac{dx_{ij}^{\alpha\beta}}{dt} = -x_{ij}^{\alpha\beta}$, and after each presynaptic spike, at time $t = t_i^* + d_{ij}^{\alpha\beta}$: $x_{ij}^{\alpha\beta} \to x_{ij}^{\alpha\beta} + g_{ij}^{\alpha\beta}$

**F: Stimulus**	
Type	Current input
Target	E1, E2
Description	$I_{\mathrm{stim},k}^\beta(t) = I_0(\underbrace{1 + c\gamma^\beta + \sigma_{\mathrm{stim}} z^\beta(t)}_{s^\beta(t)} + \underbrace{\sigma_{\mathrm{ind},k} z_k^\beta(t)}_{s_k^\beta(t)})$ during the stimulus interval and 0 otherwise. $z^\beta(t)$ and $z_k^\beta(t)$ are independent realizations of an Ornstein-Uhlenbeck process, defined by $\tau_{\mathrm{stim}} \frac{dz}{dt} = -z + \sqrt{2\tau_{\mathrm{stim}}}\xi(t)$, where $\xi(t)$ is Gaussian white noise (mean 0, variance dt).

G: Measurements

Spikes from all neurons of populations E1 and E2.

Supplementary Table 2. Simulation parameters for the sensory circuit model.

**Populations**		
$N_{E1} = N_{E2}$	800	Size of excitatory populations E1 and E2
N_I	400	Size of inhibitory population I
N_X	1000	Size of external population X
**Connectivity]**		
Local recurrent connections		
p	0.2	Connection probability for (EE, EI, IE, II)
w_+	1.3	Relative synaptic strength of connections within populations E1 and E2
w_-	$2 - w_+ = 0.7$	Relative synaptic strength of connections across populations E1 and E2
$\bar{g}_{E1E1} = \bar{g}_{E2E2}$	$0.76\ \text{nS} \cdot w_+$	Weight of excitatory to excitatory synapses within populations E1 and E2
$\bar{g}_{E1E2} = \bar{g}_{E2E1}$	$0.76\ \text{nS} \cdot w_-$	Weight of excitatory to excitatory synapses across populations E1 and E2
$\bar{g}_{EI}$	1.52 nS	Weight of excitatory to inhibitory synapses
$\bar{g}_{IE} = \bar{g}_{II}$	12.6 nS	Weight of inhibitory to excitatory (inhibitory) synapses
$\sigma_{\alpha\beta}$	$0.5 \cdot \bar{g}_{\alpha\beta}$	Standard deviation of synaptic weights from neurons in population α to neurons in population β
d_E	[0.5 ms, 1.5 ms]	Range of uniformly distributed transmission delays of excitatory connections
d_I	[0.1 ms, 0.9 ms]	Range of uniformly distributed transmission delays of inhibitory connections
External connections		
p_x	0.32	Connection probability for external connections
$\bar{g}_{XE} = \bar{g}_{XI}$	1.71 nS	Weight of external to excitatory (inhibitory) synapses
$\sigma_{X\beta}$	$0.5 \cdot \bar{g}_{X\beta}$	Standard deviation of synaptic weights from neurons in external population to neurons in population β
d_XE	[0.5 ms, 1.5 ms]	Range of uniformly distributed conduction delays of external connections
Feedback connections from the decision circuit		
p_FB	0.2	Connection probability for feedback connections
b_FB	varied	Feedback strength in the homogeneous network
g_FB	$0.0668\,\text{nS} \cdot b_\text{FB}$	Synaptic weight of feedback connections to neuron k from the corresponding population of the decision circuit (D1 → E1, D2 → E2)
d_FB	1 ms	Fixed synaptic delay / conduction delay of feedback connections
**Neuron model**		
C_m	250 pF	Membrane capacitance
g_L	16.7 nS	Leak conductance
V_L	–70 mV	Resting potential
V_T	–50 mV	Spiking threshold
V_R	–60 mV	Reset potential
τ_ref^E	2 ms	Absolute refractory period of excitatory neurons
τ_ref^I	1 ms	Absolute refractory period of inhibitory neurons
V_{init}	[–48 mV, -50 mV]	Range of initial uniform distribution of membrane potentials
ν_{ext}	12.5 spikes/s	Firing rate of external Poisson neurons
**Synapse model**		
V_rev^E	0 mV	Reversal potential of excitatory synapses
V_rev^I	–80 mV	Reversal potential of inhibitory synapses
τ_decay	5 ms	Decay constants of AMPA-type and GABA-type conductances
τ_rise	1 ms	Rise constant of AMPA- and GABA-type conductances
**Stimulus**		
I_0	0.08 nA	Mean input current for zero-coherence stimulus
c	varied	Stimulus coherence (between 0 and 1)
γ^{E1}	+0.25	Average additional input current to E1 at highest coherence (c = 1)
γ^{E2}	–0.25	Average additional input current to E2 at highest coherence (c = 1)
σ	varied	Amplitude of temporal modulations of the stimulus
σ_stim	$0.212 \cdot \sigma$	S.d. of modulations of stimulus inputs $s^\beta(t)$ in the homogeneous network
σ_ind	$0.212 \cdot \sigma$	S.d. of modulations in individual inputs $s_k^\beta(t)$ in the homogeneous network
τ_stim	20 ms	Correlation time constant of Ornstein-Uhlenbeck process

Supplementary Table 3. Tabular description of the integration circuit model.

**A: Model Summary**	
Populations	Three excitatory, one inhibitory
Connectivity	All-to-all connections
Neuron model	Leaky integrate-and-fire, fixed voltage threshold, fixed absolute refractory time
Synapse model	Conductance-based inputs (exponentially decaying PSC)
Input	Independent fixed-rate Poisson spike trains to each neuron;<br>Feed-forward signal from the sensory circuit to excitatory neurons
Measurements	Spiking activity

**B: Populations**		
**Name**	**Elements**	**Size**
D1	Iaf neuron	$N_{E1} = 4\ N_I f_E$
D2	Iaf neuron	$N_{E2} = 4\ N_I f_E$
Dn	Iaf neuron	$N_{EN} = 4\ N_I (1\text{-}2f_E)$
I	Iaf neuron	N_I

**C: Connectivity**			
**Name**	**Source**	**Target**	**Pattern**
<u>*Local recurrent connections*</u>			
DiDj	Di	Dj	All-to-all, fixed weights $g^{\mathrm{DiDj}}_{\mathrm{AMPA}}$ and $g^{\mathrm{DiDj}}_{\mathrm{NMDA}}$, fixed delay d
DiI	Di	I	All-to-all, fixed weights $g^{\mathrm{DiI}}_{\mathrm{AMPA}}$ and $g^{\mathrm{DiI}}_{\mathrm{NMDA}}$, fixed delay d
IDj	I	Dj	All-to-all, fixed weights $g^{\mathrm{IDj}}_{\mathrm{GABA}}$, fixed delay d
II	I	I	All-to-all, fixed weights $g^{\mathrm{II}}_{\mathrm{GABA}}$, fixed delay d
<u>*Feed-forward connections from the sensory circuit*</u>			
FF	Ei (sensory)	Di (decision)	Random with sparseness p_{FF} , fixed weight $g^{\mathrm{FF}}_{\mathrm{AMPA}}$, delay d_{FF}

**D: Neuron models**	
Name	Iaf neuron
Type	Leaky integrate-and-fire, conductance input
Subthreshold dynamics	$C^\beta_m \frac{dV^\beta_j}{dt} = -g^\beta_L\left(V^\beta_j - V_L\right) + I^{\alpha\beta}_{\mathrm{AMPA},j}(t) + I^{\alpha\beta}_{\mathrm{NMDA},j}(t) + I^{\alpha\beta}_{\mathrm{GABA},j}(t) + I^{\mathrm{EXT},\beta}_{\mathrm{AMPA},j}(t) + I^{\mathrm{FF},\beta}_{\mathrm{AMPA},j}(t)$, for $t > t^* + \tau^\beta_{\mathrm{ref}}$<br>$V^\beta_j(t) = V_R$, otherwise
Synaptic currents	$I^{\alpha\beta}_{\mathrm{AMPA},j}(t) = -\sum_i s^{\alpha\beta}_{\mathrm{AMPA},ij}\left(V^\beta_j - V^\alpha_{\mathrm{rev}}\right)$, where i runs over all presynaptic neurons from population α<br>$I^{\alpha\beta}_{\mathrm{NMDA},j}(t) = -1/(1+\exp(-0.062\ \mathrm{mV}^{-1} \cdot V^\beta_j)/3.57) \cdot \sum_i s^{\alpha\beta}_{\mathrm{NMDA},ij}\left(V^\beta_j - V^\alpha_{\mathrm{rev}}\right)$<br>$I^{\alpha\beta}_{\mathrm{GABA},j}(t) = -\sum_i s^{\alpha\beta}_{\mathrm{GABA},ij}\left(V^\beta_j - V^\alpha_{\mathrm{rev}}\right)$<br>$I^{\mathrm{EXT},\beta}_{\mathrm{AMPA},j}(t) = -s^{\mathrm{EXT}\beta}_{\mathrm{AMPA},j}\left(V^\beta_j - V^\alpha_{\mathrm{rev}}\right)$<br>$I^{\mathrm{FF},\beta}_{\mathrm{AMPA},j}(t) = -\sum_i s^{\mathrm{FF},\beta}_{\mathrm{AMPA},ij}\left(V^\beta_j - V^\alpha_{\mathrm{rev}}\right)$, where i runs over all presynaptic neurons from the *sensory* population α
Spiking	if $V^\beta_j(t^-) < V_T$ and $V^\beta_j(t^+) \geq V_T$: (1) set $t^* = t$ and (2) emit spike with time stamp t^*

**E: Synapse model**	
**Type**	**Synaptic dynamics**
AMPA synapse	$\tau_{\mathrm{AMPA}} \frac{ds^{\alpha\beta}_{\mathrm{AMPA},ij}}{dt} = -s^{\alpha\beta}_{\mathrm{AMPA},ij}$, and after each presynaptic spike, at time $t = t^*_i + d$: $s^{\alpha\beta}_{\mathrm{AMPA},ij} \to s^{\alpha\beta}_{\mathrm{AMPA},ij} + g^{\alpha\beta}_{\mathrm{AMPA}}$
GABA synapse	$\tau_{\mathrm{GABA}} \frac{ds^{\alpha\beta}_{\mathrm{GABA},ij}}{dt} = -s^{\alpha\beta}_{\mathrm{GABA},ij}$, and after each presynaptic spike, at time $t = t^*_i + d$: $s^{\alpha\beta}_{\mathrm{GABA},ij} \to s^{\alpha\beta}_{\mathrm{GABA},ij} + g^{\alpha\beta}_{\mathrm{GABA}}$
NMDA synapse	$\frac{ds^{\alpha\beta}_{\mathrm{NMDA},ij}}{dt} = -\frac{s^{\alpha\beta}_{\mathrm{NMDA},ij}}{\tau_{\mathrm{NMDA,decay}}} + \alpha_{\mathrm{NMDA}} \cdot x^{\alpha\beta}_{ij} \cdot \left(1 - s^{\alpha\beta}_{\mathrm{NMDA},ij}\right)$<br>$\tau_{\mathrm{NMDA,rise}} \frac{dx^{\alpha\beta}_{ij}}{dt} = -x^{\alpha\beta}_{ij}$, and after each presynaptic spike, at time $t = t^*_i + d$: $x^{\alpha\beta}_{ij} \to x^{\alpha\beta}_{ij} + g^{\alpha\beta}_{\mathrm{NMDA}}$

**F: Inputs**	
Type	Poisson generator
Target	All neurons (in D1, D2, Dn, I)
Description	Spikes are emitted according to a Poisson process with rate ν^β_{EXT} independent for each neuron and mediated by AMPA synapses with weight $g^{\mathrm{EXT},\beta}_{\mathrm{AMPA}}$.

**G: Measurements**
Spikes from all neurons of populations E1 and E2.

Supplementary Table 4. Simulation parameters for the integration circuit model.

**Populations**		
f_E	0.15	Fraction of stimulus-selective excitatory neurons
$N_{D1} = N_{D2}$	240	Size of excitatory populations D1 and D2
N_{DN}	1120	Size of excitatory population Dn
N_I	400	Size of inhibitory population I
**Connectivity]**		
Local recurrent connections		
w_+	1.6	Relative recurrent synaptic strength within populations D1 and D2
w_-	$1 - \frac{f_E\ (w_+ - 1)}{(1 - f_E)}$	Relative recurrent synaptic strength of connections across populations D1 and D2 and from the non-selective population Dn to D1 and D2
$g_{\mathrm{AMPA}}^{\mathrm{D1D1}} = g_{\mathrm{AMPA}}^{\mathrm{D2D2}}$	$0.05\ \mathrm{nS} \cdot w_+$	Weight of AMPA synapses within populations D1 and D2
$g_{\mathrm{NMDA}}^{\mathrm{D1D1}} = g_{\mathrm{NMDA}}^{\mathrm{D2D2}}$	$0.165\ \mathrm{nS} \cdot w_+$	Weight of NMDA synapses within populations D1 and D2
$g_{\mathrm{AMPA}}^{\alpha\mathrm{Dn}}$	0.05 nS	Weight of AMPA synapses to neurons in Dn
$g_{\mathrm{NMDA}}^{\alpha\mathrm{Dn}}$	0.165nS	Weight of NMDA synapses to neurons in Dn
$g_{\mathrm{AMPA}}^{\alpha\mathrm{D1}} = g_{\mathrm{AMPA}}^{\alpha\mathrm{D2}}$	$0.05\ \mathrm{nS} \cdot w_-$	Weight of AMPA synapses D2 → D1, Dn → D1, D1 → D2, and Dn → D2
$g_{\mathrm{NMDA}}^{\alpha\mathrm{D1}} = g_{\mathrm{NMDA}}^{\alpha\mathrm{D2}}$	$0.165\ \mathrm{nS} \cdot w_-$	Weight of NMDA synapses D2 → D1, Dn → D1, D1 → D2, and Dn → D2
$g_{\mathrm{AMPA}}^{\mathrm{DI}}$	0.04 nS	Weight of excitatory to inhibitory synapses (AMPA)
$g_{\mathrm{NMDA}}^{\mathrm{DI}}$	0.13 nS	Weight of excitatory to inhibitory synapses (NMDA)
$g_{\mathrm{GABA}}^{\mathrm{ID}}$	1.3 nS	Weight of inhibitory to excitatory synapses
$g_{\mathrm{GABA}}^{\mathrm{II}}$	1.0 nS	Weight of inhibitory to inhibitory synapses
d	0.5 ms	Transmission delay of recurrent excitatory and inhibitory connections
External connections and feed-forward connections from the sensory circuit		
$g_{\mathrm{AMPA}}^{\mathrm{EXT,D}}$	2.1 nS	Weight of external input to excitatory neurons
$g_{\mathrm{AMPA}}^{\mathrm{EXT,I}}$	1.62 nS	Weight of external input to inhibitory neurons
p_{FF}	0.2	Connection probability for feedback connections
$g_{\mathrm{AMPA}}^{\mathrm{FF}}$	0.09 nS	Synaptic weight of feed-forward connections from the corresponding stimulus-encoding population from the sensory circuit (E1 → D1, E2 → D2)
d_{FF}	1 ms	Transmission delay of feed-forward connections
**Neuron model**		
$C_{\mathrm{m}}^{\mathrm{e}}$	500 pF	Membrane capacitance of excitatory neurons
$C_{\mathrm{m}}^{\mathrm{i}}$	250 pF	Membrane capacitance of inhibitory neurons
g_L^e	25 nS	Leak conductance of excitatory neurons
g_L^i	20 nS	Leak conductance of inhibitory neurons
V_L	–70 mV	Resting potential
V_T	–50 mV	Spiking threshold
V_R	–55 mV	Reset potential
$\tau_{\mathrm{ref}}^{\mathrm{e}}$	2 ms	Absolute refractory period of excitatory neurons
$\tau_{\mathrm{ref}}^{\mathrm{i}}$	1 ms	Absolute refractory period of inhibitory neurons
V_{init}	[–48 mV, –50 mV]	Range of initial uniform distribution of membrane potentials
**Synapse model**		
V_{rev}^{E}	0 mV	Reversal potential of excitatory synapses
V_{rev}^{I}	–70 mV	Reversal potential of inhibitory synapses
τ_{AMPA}	2 ms	Decay constant of AMPA-type conductances
τ_{GABA}	5 ms	Decay constant of GABA-type conductances
$\tau_{\mathrm{NMDA,decay}}$	100 ms	Decay constant of NMDA-type conductances
$\tau_{\mathrm{NMDA,rise}}$	2 ms	Rise constant of NMDA-type conductances
α_{NMDA}	0.5 ms^{-1}	Saturation constant of NMDA-type conductances
**Inputs**		
$\nu_{\mathrm{EXT}}^{\mathrm{D1}} = \nu_{\mathrm{EXT}}^{\mathrm{D2}}$	2392 spikes/s	Firing rate of external Poisson input to neurons in population D1 and D2
$\nu_{\mathrm{EXT}}^{\mathrm{En}} = \nu_{\mathrm{EXT}}^{\mathrm{I}}$	2400 spikes/s	Firing rate of external Poisson input to neurons in population Dn and I

Supplementary Methods

Network model

A standardized description of the model and all simulation parameters can be found in **Supplementary Tables 1-4**[11,12].

Choice bias: The random connectivity within the sensory circuit and across circuits (i.e. bottom-up and top-down) can cause that the network's behavioral responses exhibit a bias towards one of the two choice options. We avoided this "quenched" bias by testing 10 different connectivity matrices and selecting the one that showed the least bias (51% choice 1 vs. 49% choice 2). This connectivity matrix was used in all simulations. Our conclusions remain valid for networks that show higher choice biases.

Stimulus model: The stimulus term $s^{\beta}(t)$ (see equation 2 in Methods) that is common to all neurons of a population and the individual inputs $s_k^{\beta}(t)$ (see equation 3 in Methods) that are independently generated for each neuron k have the same standard deviation $\sigma_{\text{stim}} = \sigma_{\text{ind},k} = 0.212 \cdot \sigma$. This yields an average correlation between the currents injected into two different neurons k and k' of $\text{CorrCoef}\left[I^{\alpha}_{\text{stim},k}(t), I^{\beta}_{\text{stim},k'}(t)\right] = 0.5$ for $\alpha = \beta$, and zero for $\alpha \neq \beta$. This stimulus parameterization, adding a common and an independent term, accounts for the fact that each MT neuron "sees" the RDK through the specific and heterogeneous receptive fields of its afferent inputs[13], causing the PSTHs from pairs of neurons obtained from repeated presentations of the replicate stimuli to be partially correlated (average correlation coefficient ~0.5, PSTH bin size T = 20 ms; **Supplementary Fig. 1b-c**). This is comparable to the two pairs in the MT data set which were recorded with replicate RDKs and exhibited different PSTHs[10] (correlation = 0.47 and 0.26 with direction difference = 9º and 20º for pairs emu035 and emu034, respectively; bin size T = 60 ms; coherence 0%). Introducing higher correlations in the current inputs to sensory neurons led to an increase in pair-wise correlations and to an increase in the early CP (**Fig. 2b,c**) but left all results unchanged (not shown).

Psychophysical data

Because the stimuli used during the MT recordings were not stored, we trained two male adult macaque monkeys (*Macaca mulatta*) to report the net motion direction of a fixed-duration random dot kinematogram (RDK, see below) along the horizontal axis with varying levels of difficulty (depending on the motion coherence of the RDK). On each trial we recorded both the monkey's choice and the presented stimulus (i.e. the positions of the dots in each frame). These data were used to compute average motion energy traces (**Supplementary Fig. 7c,e**). Minor stimulus differences aside (see above), the task was very similar to the classical fixed-duration version[7,8,14], except that: (1) the monkey reported his choice with a reaching response rather than with a saccadic eye movement, (2) the targets were displayed 500 ms before stimulus onset rather than only after stimulus offset. We are confident that these differences do not provide a reason for expecting a different dynamics of decision formation (reflected in a different psychophysical kernel). Importantly, and as in the original studies, the monkeys were only trained in the fixed-duration task. They were never exposed to other variants of the task, which could in principle encourage the decision to be formed rapidly (such as reaction time tasks or variable duration tasks). All surgical and behavioral procedures conformed to guidelines established by the National Institutes of Health and were approved by the Institutional Animal Care and Use Committee of Stanford University.

Random dot kinematograms

The stimulus used in our psychophysical experiment – a random dot kinematogram (RDK)[14] – was generated using MATLAB and Psychophysics Toolbox[15]. Stimuli were presented on a 19-inch LCD touch monitor (Elo Touchsystems) with 75 Hz frame rate and 800 x 600 pixels resolution positioned 30 cm away from the monkey. The details for generating the stimulus have been extensively described previously[5,16]. We used the same algorithm and parameters expect: (1) the

stimulus duration was fixed at 1 s, (2) the diameter of the stimulus aperture was 14 degrees, and (3) the speed of the dots was 8 degrees / second. Otherwise, we used the same coherences (0, 3.2, 6.4, 12.8, 25.6, and 51.2%), dot density (16.7 dots deg^{-2} s^{-1}), and dot size (2 pixels). To create the impression of motion, the dots in the RDK were split into 3 consecutive sets of the same size (1 set displayed for each individual frame) and displaced 3 frames (40 ms) later. The fraction of dots displaced coherently toward one of the two targets was determined by the coherence (motion strength), with the remaining dots being displaced randomly. For zero-coherence stimuli all dots were displaced randomly but, due to the stochasticity of that process, one obtains non-zero net motion toward the targets over a small number of frames (i.e. the motion energy shows temporal modulations).

The MT data[7,8,14] analyzed here was obtained using 2-second RDK stimuli, which varied from session to session to match the stimulus preference of the MT neuron under study (diameter of the stimulus, the speed of the dots, between 0.4 and 28.4 degrees/second, and the axis of motion). The algorithm used to generate the stimulus was also different[14]. Despite these differences in stimuli and task structure, the monkeys' behavioral performance was similar across all studies (including ours, see **Supplementary Fig. 7b,d,f**).

Psychophysical reverse correlation

We used psychophysical reverse correlation[17,18] in order to measure the amplitude and time-course of the impact of stimulus fluctuations on the decision. The psychophysical kernels were computed as the difference of the average stimulus leading to each of the two possible choices. For the network model, it was defined as:

$$PK(t) = z_1(t) - z_2(t)\,, \qquad \textbf{(1)}$$

where $z_\alpha(t) = \left\langle z^l(t) \right\rangle_{l\in\alpha}$ is the average of the stimulus fluctuations across trials yielding choice α (α =1,2). The stimulus fluctuations in each trial were defined as $z^l(t) = z^{\mathrm{E1},l}(t) - z^{\mathrm{E2},l}(t)$, where $z^{\beta,l}(t)$ are the temporal modulations in sensory input into population β in trial l (see equation (2)). Psychophysical kernels were smoothed by convolution with a 100 ms rectangular window.

The *sensory integration window* is defined as the interval, starting at stimulus onset, which contains 85% of the total area under the psychophysical kernel. The length of this window quantifies the period in which the system is sensitive to the sensory input.

To compute the psychophysical kernel from the experimental data (**Supplementary Fig. 7c,e**) we used equation 1, but defined the stimulus fluctuations in each trial $z^l(t)$ as the difference of the motion energies[6] in the two directions that the monkey had to discriminate (left vs. right)[5]: $z^l(t) = m_L^l(t) - m_R^l(t)$. Oriented motion energy $m_\alpha^l(t)$ in direction α (α = L, R) and trial l was calculated as a spatial average of the motion energy $m_\alpha^l(\mathrm{x},t)$ defined at each pixel $\mathbf{x} = (x,y)$. This was computed as the spatiotemporal convolution of a quadrature pair of oriented filters, $F_\alpha(\mathrm{x},t)$ and $F'_\alpha(\mathrm{x},t)$, and the stimulus image $I^l(\mathbf{x},t)$ (i.e. the random dot pattern in each frame t):

$$m_\alpha^l(\mathrm{x},t) = \left[F_\alpha(\mathrm{x},t) * I^l(\mathrm{x},t)\right]^2 + \left[F'_\alpha(\mathrm{x},t) * I^l(\mathrm{x},t)\right]^2 .$$

The filters were implemented as in ref. [5]. The space constants σ_c and σ_g were multiplied by a factor 2.8 in order to adjust the spatiotemporal frequency passband to the speed of coherent motion which was higher in the current experiment (7.95 degrees / second vs. 2.84 degrees / second).

Statistical methods

We mainly used permutation tests to establish the statistical significance of our results. Paired tests were used to compare measurements within neurons, implemented by permutation of trials across different conditions. When performing two-sample tests (non-paired tests), we permuted neurons across the two independent samples conserving their sample size. When the model prediction identified the sign of the comparison we used one-sided tests, and this is explicitly indicated in the text. In particular, we used the following procedures for the permutation tests: Significant deviation of CP and psychophysical kernel from chance level (**Supplementary Fig. 7**) was tested by permutation of the choice outcome in each trial. Significant difference between replicate and non-replicate conditions were tested by shuffling neurons (which were either recorded with replicate or non-replicate stimuli) between the two conditions (Fano factors and CPs, **Fig. 4**). For the subset of neurons for which both conditions were available, we instead shuffled trials between the conditions within each neuron . The same was done for the spike count correlations of the two available pairs (**Fig. 4b** and **Supplementary Fig. 8b**). Positive slope of the regression of CP correlations (**Fig. 5f**) was tested using permutations of the time stamps of CP measurements of individual neurons, recalculating the correlations and their slope for the shuffled data. Similarly, the positive slope of lagged correlations (**Fig. 6g**) was tested by individually shuffling the time stamps of lagged correlations for each neuron and recalculating the slope for the shuffled data. Standard errors were calculated using bootstrap, based on 1,000 samples obtained by randomly resampling with replacement.

When applying parametric tests (ANOVA , *t*-test), we verified that our data satisfied the assumptions of normality and homoscedasticity (Lilliefors test of normality, $P > 0.05$, and Levene's test for equality of variances, $P > 0.05$, respectively). Paired tests are used to compare measurements within neurons, in ANOVAs this is accomplished by using a mixed-effects design with neuron as random factor, nested in the monkey factor. In all analyses, outliers beyond 3 standard deviations of the population mean were removed for population tests and descriptive statistics are indicated by mean ± s.e.m.

References

1. Rocha, J. de la, Doiron, B., Shea-Brown, E., Josić, K. & Reyes, A. Correlation between neural spike trains increases with firing rate. *Nature* **448,** 802–806 (2007).
2. Polk, A., Litwin-Kumar, A. & Doiron, B. Correlated neural variability in persistent state networks. *Proc. Natl. Acad. Sci. U. S. A.* **109,** 6295–6300 (2012).
3. Shadlen, M. N., Britten, K. H., Newsome, W. T. & Movshon, J. A. A computational analysis of the relationship between neuronal and behavioral responses to visual motion. *J Neurosci* **16,** 1486–1510 (1996).
4. Cain, N. & Shea-Brown, E. Impact of correlated neural activity on decision-making performance. *Neural Comput.* **25,** 289–327 (2013).
5. Kiani, R., Hanks, T. D. & Shadlen, M. N. Bounded integration in parietal cortex underlies decisions even when viewing duration is dictated by the environment. *J Neurosci* **28,** 3017–3029 (2008).
6. Adelson, E. H. & Bergen, J. R. Spatiotemporal energy models for the perception of motion. *J Opt Soc Am A* **2,** 284–299 (1985).
7. Britten, K. H., Newsome, W. T., Shadlen, M. N., Celebrini, S. & Movshon, J. A. A relationship between behavioral choice and the visual responses of neurons in macaque MT. *Vis Neurosci* **13,** 87–100 (1996).
8. Zohary, E., Shadlen, M. N. & Newsome, W. T. Correlated neuronal discharge rate and its implications for psychophysical performance. *Nature* **370,** 140–143 (1994).
9. Bair, W. & Koch, C. Temporal precision of spike trains in extrastriate cortex of the behaving macaque monkey. *Neural Comput* **8,** 1185–1202 (1996).
10. Bair, W., Zohary, E. & Newsome, W. T. Correlated firing in macaque visual area MT: time scales and relationship to behavior. *J Neurosci* **21,** 1676–1697 (2001).
11. Nordlie, E., Gewaltig, M.-O. & Plesser, H. E. Towards reproducible descriptions of neuronal network models. *PLoS Comput Biol* **5,** e1000456 (2009).
12. Kunkel. Limits to the development of feed-forward structures in large recurrent neuronal networks. *Front. Comput. Neurosci.* (2010). doi:10.3389/fncom.2010.00160
13. DeAngelis, G. C., Ghose, G. M., Ohzawa, I. & Freeman, R. D. Functional micro-organization of primary visual cortex: receptive field analysis of nearby neurons. *J Neurosci* **19,** 4046–4064 (1999).

14. Britten, K. H., Shadlen, M. N., Newsome, W. T. & Movshon, J. A. The analysis of visual motion: a comparison of neuronal and psychophysical performance. *J Neurosci* **12,** 4745–4765 (1992).
15. Pelli, D. G. The VideoToolbox software for visual psychophysics: transforming numbers into movies. *Spat. Vis.* **10,** 437–442 (1997).
16. Gold, J. I. & Shadlen, M. N. The influence of behavioral context on the representation of a perceptual decision in developing oculomotor commands. *J. Neurosci.* **23,** 632–651 (2003).
17. Neri, P. & Levi, D. M. Receptive versus perceptive fields from the reverse-correlation viewpoint. *Vision Res.* **46,** 2465–2474 (2006).
18. Nienborg, H. & Cumming, B. G. Decision-related activity in sensory neurons reflects more than a neuron's causal effect. *Nature* **459,** 89–92 (2009).
